# Phytochemical Profiles of *Artemisia albida* Willd.: Potential for PTP1B, α-Glucosidase, BNA, DPPH and Molecular Docking Analysis

**DOI:** 10.3390/molecules31213975

**Published:** 2026-11-04

**Authors:** Janar Jenis, Ayaulym Minkayeva, Chunping Tang, Changqiang Ke, Aizhamal Baiseitova, Xiaofei Shang, Haji Akber Aisa, Yang Ye

**Affiliations:** 1 University of Chinese Academy of Sciences, Beijing 100049, China; janarjenis@kaznu.kz; 2 Natural Product Research Center, Shanghai Institute of Materia Medica, Chinese Academy of Sciences, Shanghai 201203, China; tangcp@simm.ac.cn (C.T.); kechangqiang@simm.ac.cn (C.K.); 3 Research Center for Medicinal Plants, Farabi University, Almaty 050040, Kazakhstan; minkaeva.a@gmail.com (A.M.); aizhabaiseitova@gmail.com (A.B.); 4 Research Institute for Natural Products & Technology, Almaty 050046, Kazakhstan; 5 Lanzhou Institute of Husbandry and Pharmaceutical Sciences, Chinese Academy of Agricultural Sciences, Lanzhou 730050, China; shangxiaofei@caas.cn; 6 Central Asian of Drug Discovery and Development, Xinjiang Technical Institutes of Physics and Chemistry, Chinese Academy of Sciences, Urumqi 830011, China; haji@ms.xjb.ac.cn; 7 China-Serbia “Belt and Road” Joint Laboratory for Natural Products and Drug Discovery, Shanghai Institute of Materia Medica, Chinese Academy of Sciences, Shanghai 201203, China

**Keywords:** *Artemisia albida*, PTP1B, α-glucosidase, BNA, ECD, molecular docking

## Abstract

Traditional herbal medicine has been an important source of therapeutic agents to treat various diseases since long ago and the genus *Artemisia* is drawing a lot of attention owing to its diversity of biologically active secondary metabolites. In the present study, our team focused on *Artemisia albida* Willd., which was collected in the Almaty region of Kazakhstan. Using various chromatographic techniques, 15 compounds were obtained. Their structures were elucidated by HRESIMS, NMR, and ECD, revealing a new-skeleton sesquiterpenoid, Albiterpene A (**1**), together with one known sesquiterpene, one lignan, one fatty acid ester, and 11 phenolic compounds. The absolute configuration of Albiterpene A was determined by the TDDFT ECD calculation. Meanwhile, the inhibitory and radical-scavenging activities of the isolated compounds were evaluated using α-glucosidase, PTP1B, BNA and DPPH assays. Among all tested compounds, arteanoflavone exhibited the most potent α-glucosidase (IC_50_ = 3.2 ± 0.07 μM) and PTP1B (IC_50_ = 6.8 ± 0.14 μM) inhibition and strong DPPH radical-scavenging activity (IC_50_ = 1.5 ± 0.03 μM), outperforming the positive controls DNJ and Trolox. Molecular docking was employed to elucidate the possible binding modes of arteanoflavone, chlorogenic acid, and caffeic acid ethyl ester against PTP1B (1NNY) and human α-glucosidase (2QM*J*). The docking scores suggested a consistent binding affinity ranking across both targets: chlorogenic acid > arteanoflavone > caffeic acid ethyl ester.

## 1. Introduction

*Artemisia* L. is a genus of small herbs and shrubs found in northern temperate regions. It belongs to the important family Compositae (Asteraceae), one of the most numerous plant groupings, which comprises about 1000 genera and over 20,000 species. Within this family, *Artemisia* is included in the tribe Anthemideae and comprises over 500 species, which are mainly found in Asia, Europe and North America [1]. The *Artemisia* genus has a wide area of usage in different fields, including pharmaceutics, landscape architecture and agriculture [2,3].

Kazakhstan, located in the heart of Central Asia, boasts rich medicinal plant resources. These plants are a valuable source of a wide range of secondary metabolites, which serve as key ingredients for pharmaceuticals, agrochemicals, flavors, fragrances, colors, biopesticides, and food additives. Consequently, medicinal plants continue to receive significant research attention due to their proven pharmacological effects, including antioxidant, antidiabetic, antitumor, anti-inflammatory, and antibacterial properties. These therapeutic benefits are attributed to the diverse bioactive secondary metabolites present in the plants, such as flavonoids, triterpenoids, steroids, alkaloids, saponins, proteins, and amino acids.

Although *Artemisia* species from Kazakhstan are a promising source of bioactive substances, they have not been investigated thoroughly. In our previous work, we aimed to identify potential active candidates, which encouraged us to analyze the protein tyrosine phosphatase 1B (PTP1B), α-glucosidase, and bacterial neuraminidase (BNA) inhibitory potentials, as well as the antioxidant activities, of nine *Artemisia* plant extracts—most of which are endemic and have not yet had their secondary metabolites and bioactivities explored [4]. Among all tested species, *A. albida*, *A. albicerata*, *A. transiliensis*, *A. nitrosa* and *A. scopaeformis* showed high potential for α-glucosidase, PTP1B, and BNA inhibition.

**Figure 1:**
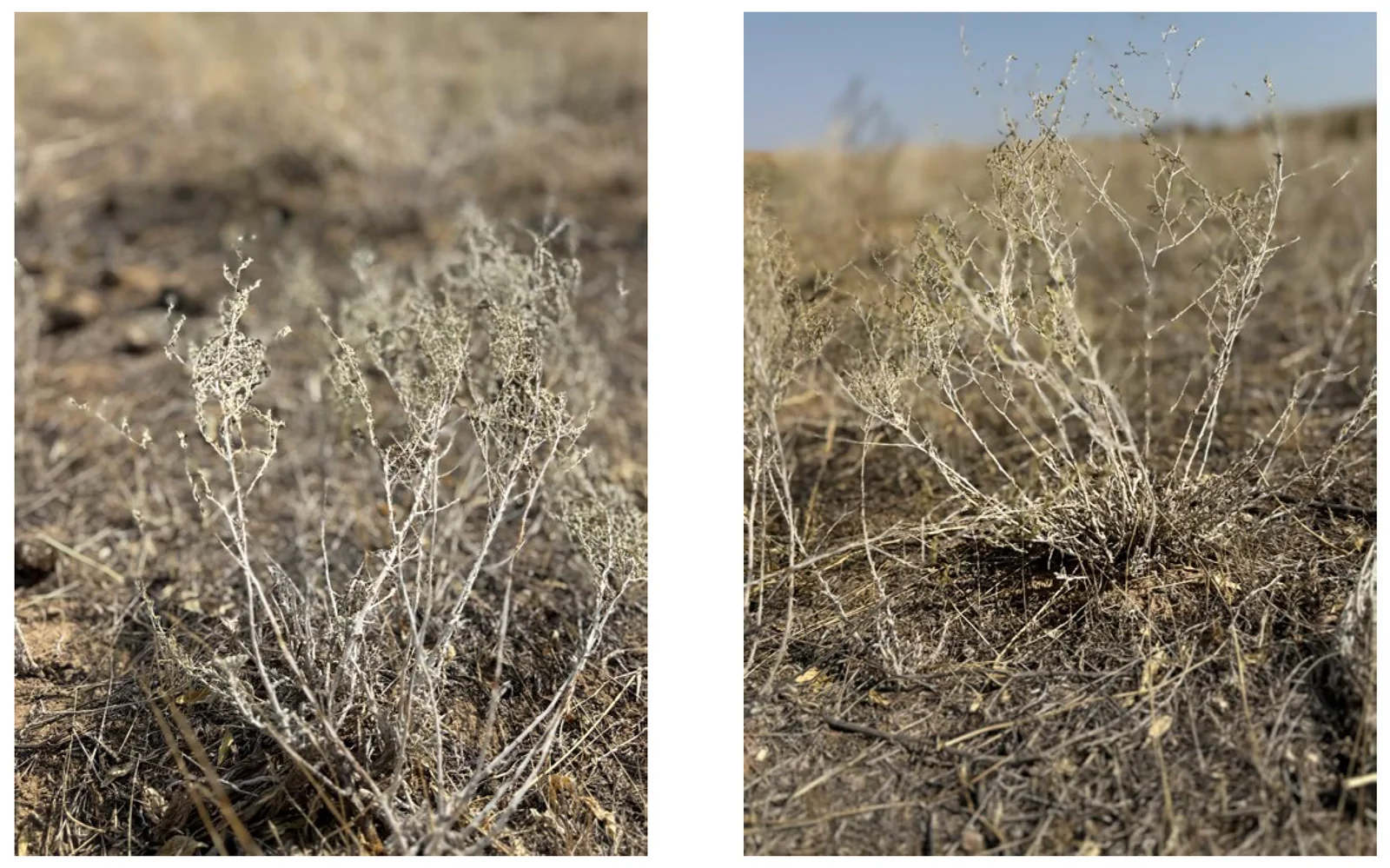
*Artemisia albida* Willd. species in its natural habitat (Almaty region, Kazakhstan). Photograph taken by Janar Jenis in end of September 2023.

*Artemisia albida* Willd., a semi-white wormwood (Figure 1) native to several regions of Kazakhstan, including Almaty, Altai, Tarbagatai, Zaysan, and Akmola [4], is a perennial plant growing in the mountains. The species is chemically rich, containing lignans, triterpenes, sesquiterpenes, alkaloids, and steroids, and therefore has considerable research potential that warrants comprehensive study. From 2005 to 2008, *A. albida* Willd., collected from the Ivanovsky ridge (Altai) in East Kazakhstan, was first studied by S. M. Adekenov and E. M. Suleimenov, who isolated eupatilin and its 5′-methyl ether, the sesquiterpene lactones austricin, matricarin, canin, and argolide, anhydroaustricin, and two eupatilin-derived flavonoids, including its 7-O-methyl ether, from the aerial part of *A. albida* [5,6]. In addition, qualitative and quantitative analyses were carried out for the first time to determine moisture, total ash, organic acids, flavonoids, and coumarins in the whole plant, together with a quantitative assessment of the total content of biologically active components [4,7,8].

Pharmacologically, the plant extract contains substances reported to improve brain activity, contractile function of the heart and skeletal muscles, nerve conduction and the action of many hormones that play an important role in nutrition, protein structure, metabolism, signal transmission, and hemostasis, as well as to enhance immunity and protect against the development of pancreatic and prostate cancer [3,4]. The MeOH extract of *A. albida* Willd. showed a high potential to inhibit α-glucosidase (55.8%), PTP1B (85.5%) and BNA (95.5%), suggesting that the plant may contain bioactive candidates against diabetes, obesity, and bacterial infections [4]. However, despite this promising bioactivity profile, no systematic phytochemical investigation of *A. albida* in this context has been reported, and the constituents responsible for these effects remain unidentified.

**Figure 2:**
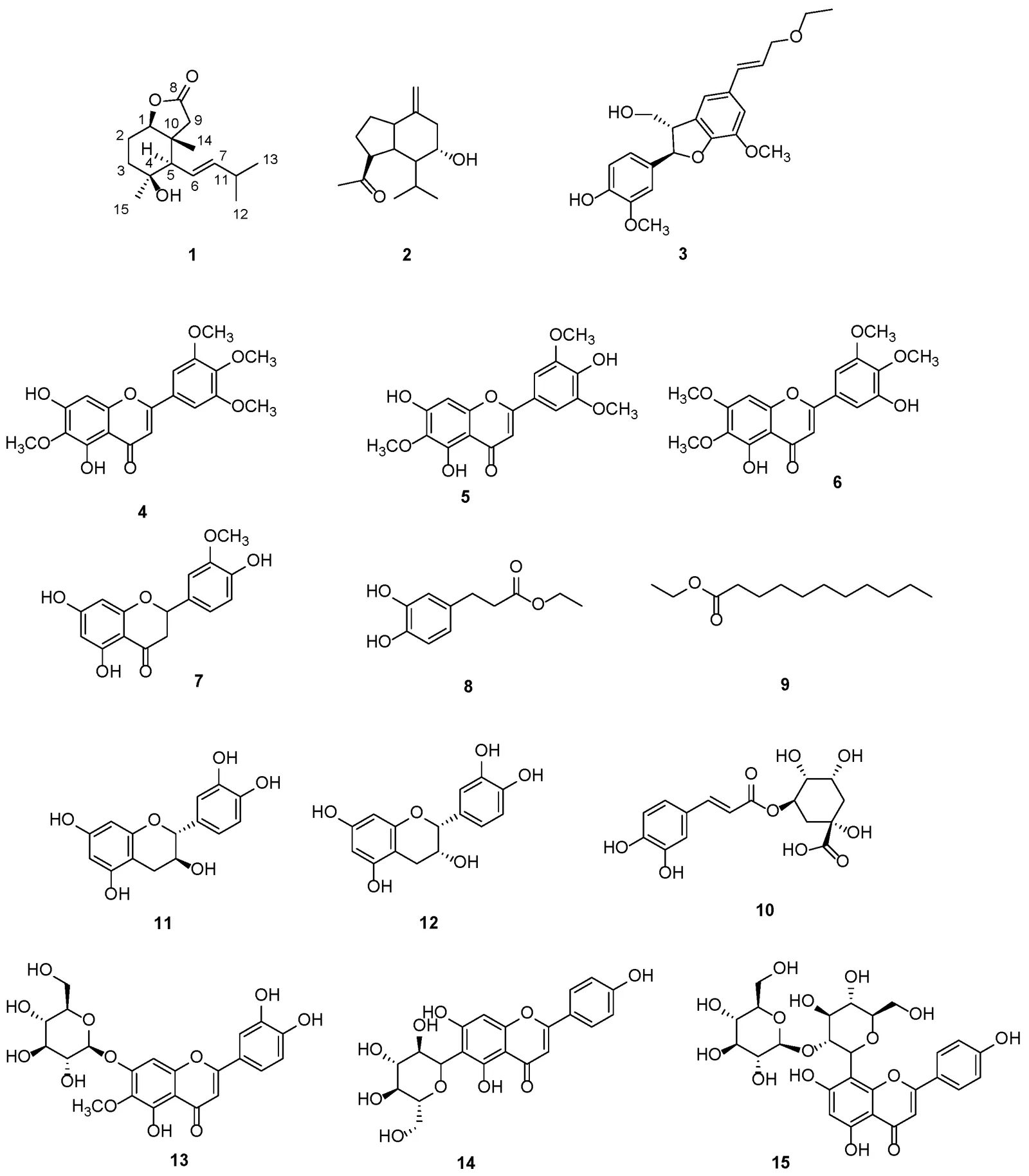
Structures of compounds (**1**–**15**) from *A. albida* Willd.

Therefore, our team focused on the chemical profiling of *A. albida* Willd., which was collected in the Almaty region of Kazakhstan, and on searching for its bioactivities. Using various chromatographic techniques—including AB-8 macroporous adsorption resin, D101 macroporous resin, Sephadex LH-20, Toyopearl HW-40F, silica gel column chromatography and semi-preparative HPLC—we isolated 15 compounds from the dichloromethane and butanol extracts. Their structures were elucidated by HRESIMS, NMR, ECD, and other methods, revealing two sesquiterpenes (**1**, **2**) (including a new-skeleton sesquiterpenoid Albiterpene A (**1**)), one lignan (**3**), one fatty acid ester (**8**), and eleven phenolic compounds (**4**–**7**, **9**–**15**) (Figure 2). All these isolates were identified from *A. albida* Willd. for the first time. Meanwhile, the bioactivity assays for the 15 compounds were carried out, followed by molecular docking analysis for selected compounds that showed significant activities against α-glucosidase, PTP1B, BNA and DPPH.

## 2. Results and Discussion

### 2.1. Isolated Compounds from A. albida

Compound **1** was obtained as a colorless amorphous powder. The molecular formula was established as C_15_H_24_O_3_ by the HRESIMS data at 275.1618 [M+Na] (275.1623, calcd for C_15_H_24_O_3_Na), corresponding to four degrees of unsaturation. The ^13^C NMR spectrum displayed 15 carbon signals, in combination with its 24 protons in the ^1^H NMR data, suggesting a sesquiterpenoid framework (Table 1). The presence of one carbonyl carbon at *δ*_C_ 179.9 (C-8) and two olefinic carbons at *δ*_C_ 142.9 (C-7) and 124.2 (C-6), corresponding to a carbonyl group and one double bond, respectively, accounted for two degrees of unsaturation. The remaining two suggested the existence of a bicyclic system, which was further supported by its 2D NMR data.

The ^1^H NMR spectrum of compound **1** displayed two downfield signals at *δ*_H_ 5.66 (1H, ddd, *J* = 15.4, 9.5, 1.2 Hz, H-6) and 5.52 (1H, dd, *J* = 15.4, 6.7 Hz, H-7). The large vicinal coupling constant (15.4 Hz) clearly defined an *E*-configured double bond. An oxymethine proton *J* at *δ*_H_ 4.01 (1H, dd, *J* = 12.5, 3.4 Hz, H-1) showed a direct HSQC correlation to an oxygen-bearing carbon at *δ*_C_ 88.6 (C-1). Another oxygenated carbon at *δ*_C_ 72.4 (C-4) displayed no direct correlation in the HSQC spectrum, indicating that it was a non-protonated tertiary carbon. Four methyl resonances were evident at *δ*_H_ 1.01 (3H, d, *J* = 6.8 Hz), 1.02 (3H, d, *J* = 6.8 Hz), 1.10 (3H, s), and 1.17 (3H, d, *J* = 1.2 Hz), which correlated in the HSQC spectrum with carbons at *δ*_C_ 23.2, 23.1, 30.7, and 14.9, respectively.

The *γ*-lactone ring and the fused cyclohexane skeleton were assembled from HMBC correlations in conjunction with the NMR features reported for the related dimethylcyclohexane-fused *γ*-lactones [9] and sesquiterpenoids from *A. annua* [10]. The oxymethine proton H-1 (*δ*_H_ 4.01) and the methylene protons H_2_-9 (*δ*_H_ 2.00 and 2.38) showed key HMBC correlations to the carbonyl carbon C-8 (*δ*_C_ 179.9), and both also correlated to the quaternary carbon C-10 (*δ*_C_ 46.5). These correlations indicated that C-1, C-8, C-9, and C-10, together with an oxygen atom, constituted a five-membered *γ*-lactone ring, in which C-1 and C-10 served as the bridgehead carbons fused to a cyclohexane ring. The cyclohexane ring was constructed from the HMBC correlations from H-1 to C-3 (*δ*_C_ 39.9), C-5 (*δ*_C_ 57.5), and C-14 (*δ*_C_ 14.9), from H_2_-2 (*δ*_H_ 1.86) to C-4, and from H-5 (*δ*_H_ 2.01) to C-3, C-14, and C-15 (*δ*_C_ 30.7), together with the ^1^H–^1^H COSY correlation of H_2_-2 and H_2_-3. The attachments of the two methyls were clearly assigned to C-10 and C-4 by HMBC correlations from Me-14 (*δ*_H_ 1.17; *δ*_C_ 14.9) to C-10, and from Me-15 (*δ*_H_ 1.10; *δ*_C_ 30.7) to C-4.

The alkenyl side chain was established by comprehensive ^1^H–^1^H COSY and HMBC analyses. In the ^1^H–^1^H COSY spectrum, H-7 showed a cross-peak with the methine proton at *δ*_H_ 2.32 (H-11), which in turn coupled with the two methyl doublets at *δ*_H_ 1.02 (Me-12) and 1.01 (Me-13), respectively. These continuous correlations, from H-6 to H-7, and then to H-11 and Me-12/Me-13, established a continuous spin system (H-6/H-7/H-11/H-12(13)), defining an aliphatic chain extending from the double bond to a terminal isopropyl group. HMBC correlations from the methine proton H-11 (*δ*_H_ 2.32) to C-12 and C-13 confirmed the attachment of the two methyl groups to C-11. Meanwhile, HMBC correlations from Me-12 and Me-13 to C-11 (*δ*_C_ 32.7) and to C-7 (*δ*_C_ 142.9) supported the isopropyl terminus located at C-7. Consequently, an *E*-3-methylbut-1-enyl side chain was defined in the molecule.

**Figure 3:**
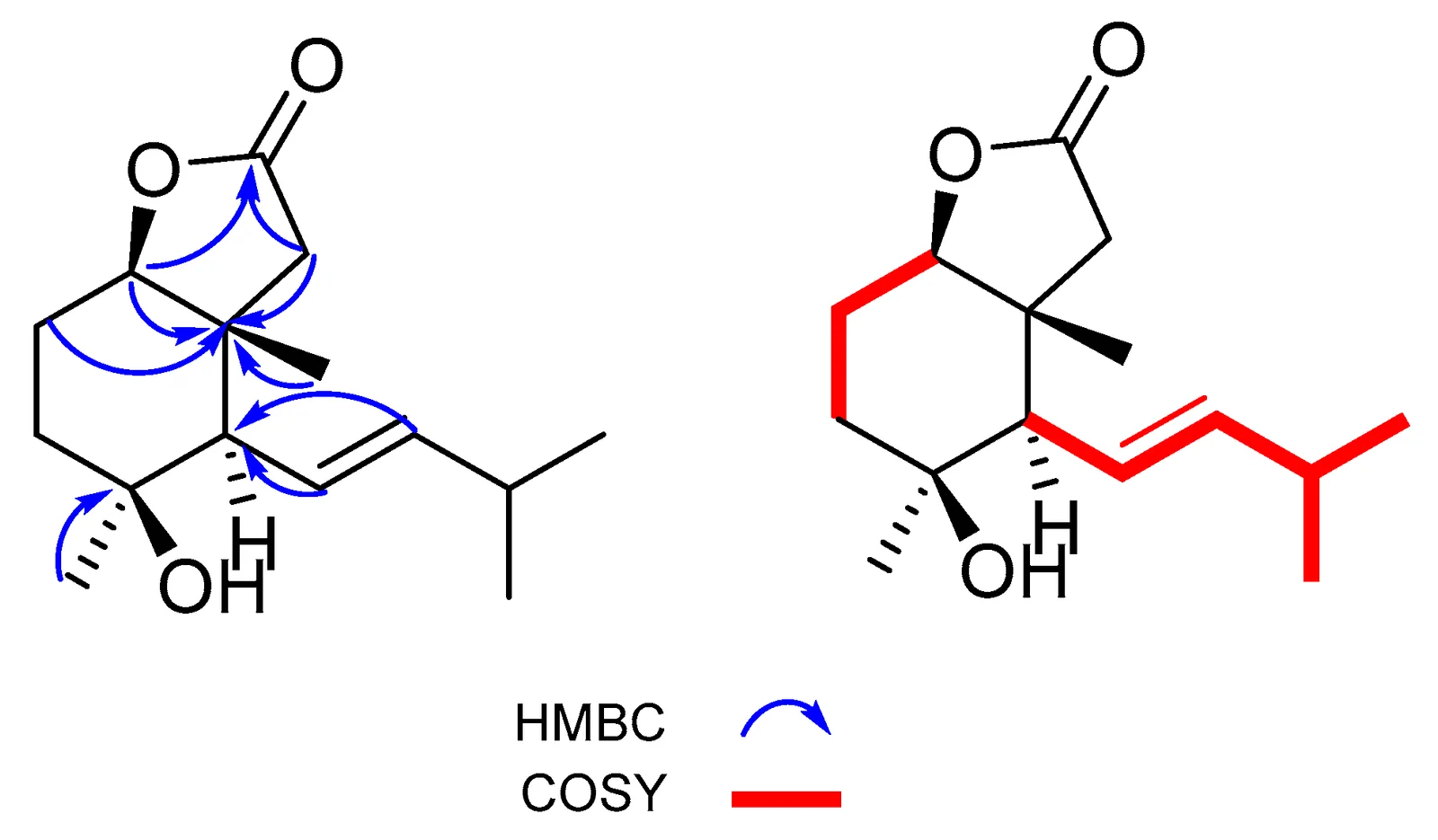
^1^H-^1^H COSY ((**right**), red bold lines) and selected HMBC correlations ((**left**), blue arrows) for compound **1**.

The attachment of this alkenyl side chain to the core ring was deduced from the critical HMBC correlations both from H-6 to C-4, C-10, and C-11, and from H-5 to C-7. These correlations unambiguously placed the side chain directly at the C-5 of the cyclohexane ring. The overall side-chain pattern is closely comparable to the side chain of seriphiloid [11], although it was established here independently from the ^1^H–^1^H COSY and HMBC data (Figure 3).

The relative configuration of compound **1** was deduced from its coupling constants and the NOESY experiment. The oxymethine proton H-1 (*δ*_H_ 4.01) appeared as a double doublet with a large coupling constant of 12.5 Hz and a smaller one of 3.4 Hz. According to the Karplus equation, the large vicinal coupling (12.5 Hz) corresponds to an axial–axial coupling constant, indicating that H-1 adopts an axial orientation on the cyclohexane ring. This arrangement implied that the lactone oxygen substituent on C-1 is equatorially oriented, which is entirely consistent with a *trans*-fused 9-oxabicyclo [4.3.0]nonane framework. At the same time, the NOESY experiment showed that cross-peaks of H-1/H-3a, H-3a/H_3_-15, H-1/H-5, and H-6/H_3_-14 were clearly observed, indicating that H-1/H-5/H_3_-15 were on the same face while H-6/H_3_-14 were on the other. Accordingly, the relative configuration of **1** was proposed as 1R*,4S*,5R*,10R*.

**Figure 4:**
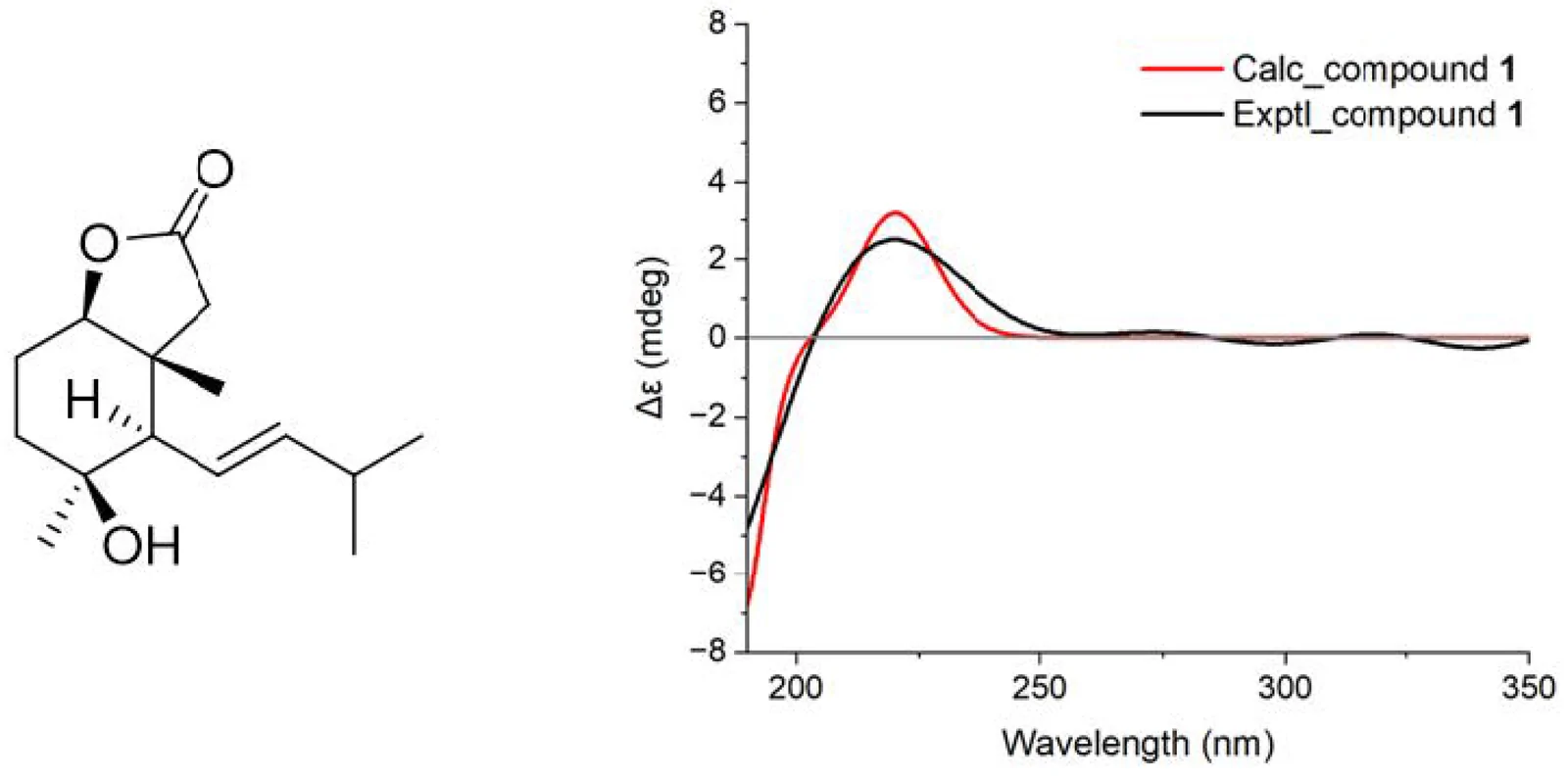
Comparison of the experimental (black) and calculated (red) ECD curves (**right**) and the candidate conformation (**left**) of compound **1**.

The absolute configuration of compound **1** was further determined by the TDDFT ECD calculation. The calculated ECD spectrum for the candidate conformation of compound **1** had a good agreement with that of the experimental curve, both showing a positive Cotton effect around 220 nm (Figure 4). Thus, the absolute configuration of **1** was determined as 1*R*,4*S*,5*R*,10*R*. Eventually, the whole structure of **1** was established, and the compound was named Albiterpene A.

**Scheme 1:**
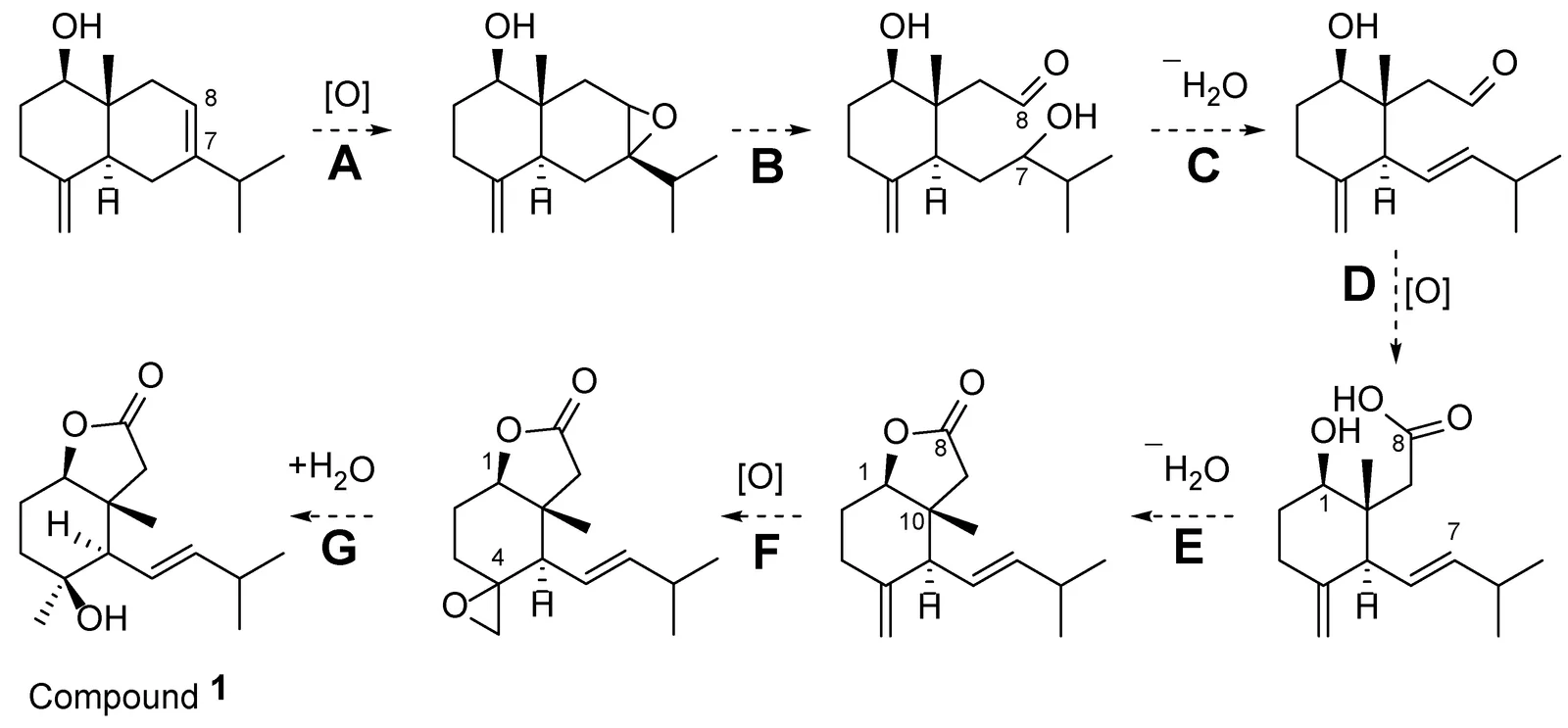
Proposed biosynthetic pathway of compound **1**.

Albiterpene A (**1**) presumably arises from oxidative processing of an eudesmane-type sesquiterpene, without the need for skeletal rearrangement (Scheme 1). A plausible co-occurring eudesmane precursor is proposed to undergo epoxidation of the Δ^7,8^ double bond (step A), followed by oxidative cleavage of the C-7–C-8 bond to open ring B (step B), affording a 7,8-seco hydroxy aldehyde; subsequent dehydration (step C) and further oxidation of the aldehyde (step D) would generate the corresponding Δ^6,7^ carboxylic acid. Intramolecular esterification between the C-8 carboxyl group and the C-1 hydroxy group (step E) then constructs the γ-lactone ring fused across the C-1/C-10 junction, thereby establishing the unusual 6/5-fused bicyclic skeleton. Finally, epoxidation of the exocyclic Δ^4(14)^ double bond (step F), followed by ring opening of the resulting spiro epoxide (a net hydration, step G), installs the tertiary C-4 hydroxy group to furnish Albiterpene A (**1**).

In addition, 14 known compounds were identified as 6α-hydroxyanhydrooplopanone (**2**) [12], (−)-(7R,8S,7′E)-4-Hydroxy-3,5′-dimethoxy-7,4′-epoxy-8,3′-neolign-7′-ene-9,9′-diol 9′-ethyl ether (**3**) [13], arteanoflavone (4) [14], 5,7,4′-tri-hydroxy-6,3′,5′-trimethoxyflavone (**5**) [15], 5,3′-dihydroxy-6,7,4′,5′-trimethoxyflavanone (**6**) [16], homoeriodictyol (**7**) [17], caffeic acid ethyl ester (**8**) [18], ethyl tridecanoate (**9**) [19], chlorogenic acid (**10**) [20], (+)-catechin (**11**) [21], epicatechin (**12**) [22], nepitrin (**13**) [23], isovitexin (**14**) [24], and vitexin-2′′-O-β-D-glucopyranoside (**15**) [25] by comparison of their NMR data with those in the literature (Figures S1–S42, Supplementary Materials).

### 2.2. The Inhibitory Potential of Isolated Constituents from A. albida Willd.

The inhibitory and radical-scavenging activities of the isolated and reference compounds were evaluated using α-glucosidase, PTP1B, BNA, and DPPH assays, and the IC_50_ values are summarized in Table 2.

**Figure 5:**
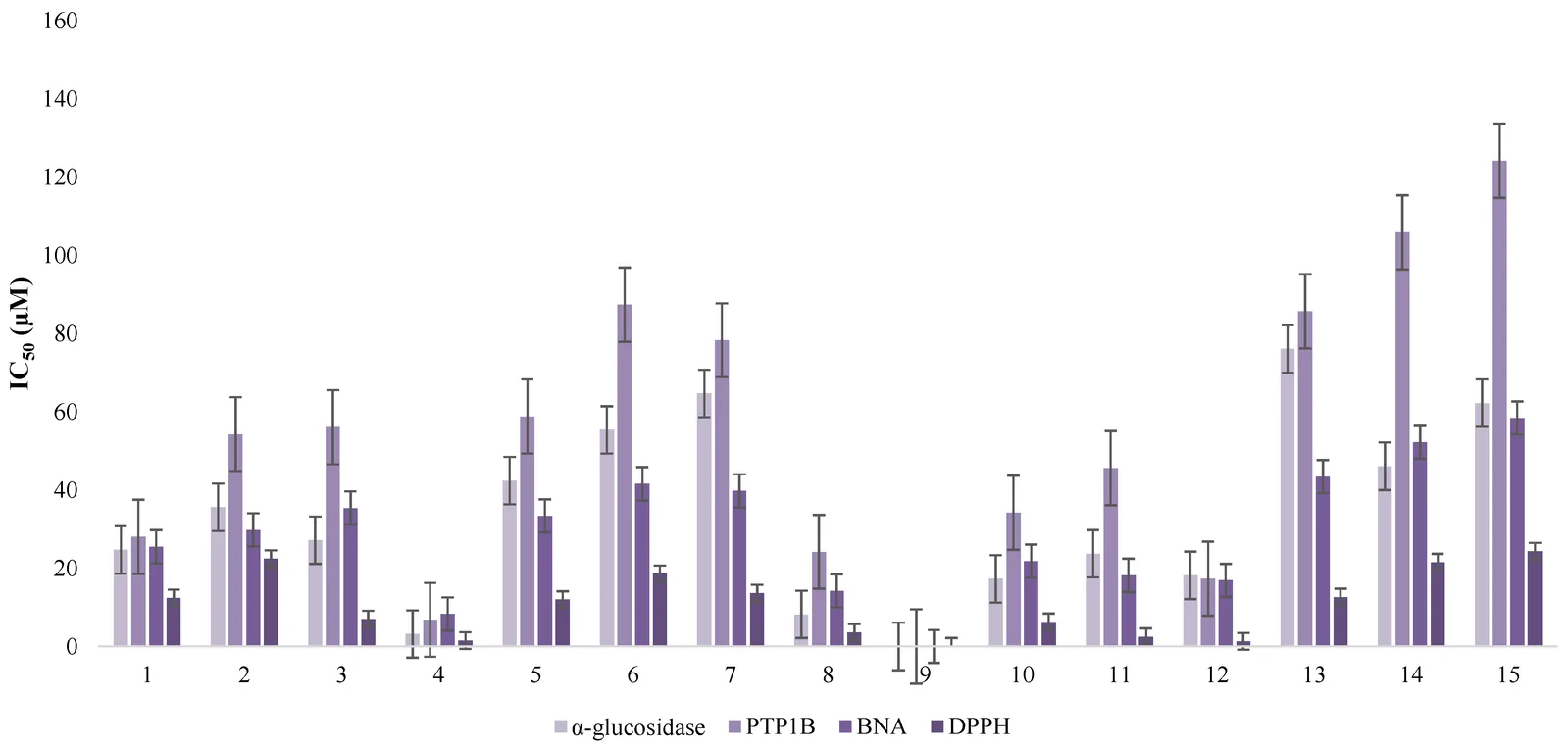
Protein Tyrosine Phosphatase 1B, α-glucosidase, bacterial neuraminidase inhibition and antioxidant potentials for 15 compounds from *A. albida* Willd.

Among all tested compounds, arteanoflavone exhibited the most potent α-glucosidase (IC_50_ = 3.2 ± 0.07 μM) and PTP1B (IC_50_ = 6.8 ± 0.14 μM) inhibition and strong DPPH radical-scavenging activity (IC_50_ = 1.5 ± 0.03 μM), outperforming the positive controls DNJ and Trolox (Figure 5). Notable dual inhibition of both α-glucosidase and PTP1B was also observed for caffeic acid ethyl ester and chlorogenic acid, consistent with the presence of catechol-type hydroxyl groups that enhance electron donation and enzyme binding through hydrogen bonding. Catechin and epicatechin demonstrated comparable multi-target activities, reflecting their well-known antioxidant and antidiabetic potential linked to the flavan-3-ol skeleton.

The newly identified sesquiterpenoid-type compound albiterpene A and 6-*α* hydroxy anhydrooplopanone showed moderate α-glucosidase (IC_50_ = 24.7 ± 0.5 and 35.6 ± 0.7 μM) and PTP1B (IC_50_ = 28.0 ± 0.6 and 54.3 ± 1.1 μM) inhibition, respectively, suggesting that the presence of hydroxyl and carbonyl moieties may contribute to enzyme interaction. However, their DPPH scavenging effects were relatively weak (IC_50_ = 12.4 ± 0.2 and 22.4 ± 0.5 μM), indicating limited electron-donating capacity compared with polyphenolic standards. Among the glycosides, vitexin-2″-O-β-D-glucopyranoside exhibited weak-to-moderate activities, possibly due to steric hindrance caused by bulky sugar moieties reducing enzyme accessibility.

Overall, flavonoid- and phenolic-type compounds demonstrated the strongest correlation between antioxidant and antidiabetic properties, emphasizing the pivotal role of hydroxylation and conjugation patterns in their bioactivity. The observed structure–activity trends indicate that polyhydroxylated and methoxylated flavonoids, as well as phenolic acids, contribute significantly to the dual inhibitory and antioxidant potential of *Artemisia*-derived metabolites.

Molecular docking was employed to elucidate the possible binding modes of arteanoflavone, chlorogenic acid, and caffeic acid ethyl ester against PTP1B (1NNY) and human α-glucosidase (2QM*J*). The Vina docking scores (Table 3) revealed a consistent binding affinity ranking across both targets: chlorogenic acid > arteanoflavone > caffeic acid ethyl ester.

It should be noted that this docking ranking does not parallel the experimental IC_50_ ranking (arteanoflavone > caffeic acid ethyl ester > chlorogenic acid). Moreover, the in vitro α-glucosidase assays were performed with the yeast enzyme, whereas 2QM*J* is the N-terminal subunit of human maltase–glucoamylase. These discrepancies may arise from scoring-function limitations and species/construct differences, and the docking results should therefore be interpreted with caution.

Against PTP1B (1NNY), chlorogenic acid exhibited the strongest binding (−7.56 kcal/mol), forming eight hydrogen bonds with key residues including Arg221, Asp181, Asp48, Gln262, Gln266, Gly183, Pro180, and Trp179. Its carboxylate moiety established salt bridges with Arg221 (4.24 Å) and Lys116. Arteanoflavone (−6.46 kcal/mol) interacted with Arg221, Asp48, and Gln266 via hydrogen bonds and displayed a face-to-face π-π stacking interaction with Tyr46 (3.75 Å, dihedral angle 13.5°). Caffeic acid ethyl ester (−5.73 kcal/mol) formed hydrogen bonds with Ala217, Gln266, Gly183, Gly220, Ser216, and Trp179, while its catechol ring participated in a cation-π interaction with Arg221 (4.09 Å), stabilized by hydrophobic packing involving Ala217, Ile219, Arg221, and Gln262 (Figure S43A–C, Supplementary Materials).

Against human α-glucosidase (2QM*J*), chlorogenic acid again demonstrated the highest affinity (−7.70 kcal/mol), forming hydrogen bonds with Arg526, Asp203, Asp443, and Asp542. It engaged in π-π stacking interactions with Phe575 and Tyr299. Arteanoflavone (−6.80 kcal/mol) formed hydrogen bonds with Arg526, Asp203, Asp327, Asp443, and Asp542, alongside T-shaped π-π stacking with Trp406 (4.92 Å) and Phe575 (5.07 Å), complemented by hydrophobic packing with Tyr299. Caffeic acid ethyl ester (−6.17 kcal/mol) established hydrogen bonds with Arg526, Asp443, and Asp542, with its binding predominantly driven by hydrophobic/π contacts involving Tyr299, Phe575 (×2), Trp406, and Gln603, and π-π stacking with Tyr299 (Figure S43D–F, Supplementary Materials).

Collectively, the docking results indicate that chlorogenic acid achieves superior predicted binding through a high density of polar contacts and salt-bridge formation, whereas arteanoflavone relies more on π-π stacking and hydrophobic interactions, and caffeic acid ethyl ester, possessing fewer polar functional groups, exhibits comparatively weaker predicted affinity. It should be emphasized that docking scores reflect calculated binding energetics only; the stronger experimental activities of arteanoflavone suggest that factors beyond simple phenolic hydroxyl density govern target engagement in vitro. Moreover, it should be noted that the molecular docking protocol applied in this work was not further validated via redocking of the corresponding crystallographic native ligand to report the RMSD reference value. Therefore, all docking poses, hydrogen bond interactions and binding mode analyses presented in this study should be inter-preted as reliable computational predictions under the current force field parameters, rather than experimentally verified atomic-level binding profiles.

## 3. Materials and Methods

### 3.1. Plant Material Collection

*Artemisia albida* Willd. was chosen for the present investigation based on the results of previous investigations and the extensive literature review. The aerial parts of the plant were gathered during the flowering phase at the end of September 2023, in the Almaty region, Kazakhstan. The confirmation of the species was done with a morphological analysis, by comparison with authenticated herbarium specimens. The voucher specimen of *A. albida* Willd. (KAZNU-230901) was deposited at the Research Center for Medicinal Plants at Al-Farabi Kazakh National University in Almaty, Kazakhstan. The collected plant material was air dried in shade, away from direct sunlight, and cut into small pieces and ground using a laboratory mill. The investigated species was thoroughly examined in respect of its phytochemical profile, isolation of secondary metabolites and its biological activity.

### 3.2. Instruments and Chemicals

All solvents and reagents used in the extraction, fractionation, isolation and purification processes were either of analytical or chemically pure grade and were used as such, unless stated otherwise. The solvents used for extractions and fractions were dichloromethane, n-Butanol, 96% ethanol, petroleum ether and acetone. For isolation of the compounds, silica gel and ODS (octadecylsilane) (Qingdao Haiyang Chemical Co., Ltd., Qingdao, China) were used as stationary phases for column chromatography and AB-8 macroporous adsorption resin (Shanghai Hualing Resin Co., Ltd., Shanghai, China), Sephadex LH-20 (Pharmacia Biotech AB, Uppsala, Sweden), Toyopearl HW-40F (Tosoh Corporation, Tokyo, Japan) were used for the adsorption and purification by size-exclusion chromatography. Materials and solvents (Sigma-Aldrich, Steinheim, Germany) were suitable for phytochemical isolation and further chemical and biological analysis.

The enzymatic activity and radical scavenging assays were carried out using a SpectraMax M3 Multi-Mode Microplate Reader (Molecular Devices, San Hoze, CA, USA). The reagents used for the assays were purchased from Sigma-Aldrich (St. Louis, MO, USA), including α-Glucosidase (EC 3.2.1.20), bacterial neuraminidase (BNA; EC 3.2.1.18), 2,2-diphenyl-1-picrylhydrazyl (DPPH), 6-hydroxy-2,5,7,8-tetramethylchroman-2-carboxylic acid (Trolox), dimethyl sulfoxide (DMSO), and others. The recombinant human protein tyrosine phosphatase 1B (PTP1B; EC 3.1.3.48) was obtained from Enzo Life Sciences. All the reagents used were of analytical grade and were suitable for biochemical and spectrophotometric analyses.

### 3.3. Extraction and Isolation

The air-dried aerial parts of *Artemisia albida* Willd (5 kg) were ground into small pieces and extracted with 70% ethanol (30 L × 3, each for 7 days) at room temperature using maceration method. The combined extracts were concentrated with a rotary evaporator at 45 °C to afford the crude extract (500 g). The crude extract was suspended in distilled water and successively partitioned 5 times each with petroleum ether, dichloromethane and butanol. The dichloromethane (DCM) fraction (51.28 g) and n-Butanol fraction (183.6 g) were selected for further phytochemical investigation.

The DCM fraction (50 g) was subjected to chromatography on an AB-8 macroporous adsorption resin column. Ethanol solutions ranging from 10% to 96% were used as a solvent system to yield ten fractions by polarity of substances, designated A1–A10.

Fraction A5 (5.7 g) was further purified by column chromatography over Sephadex LH-20 using methanol as the mobile phase, affording nine subfractions (A–I). Subfractions C, D, and E were combined together and renamed Z according to results of LC-MS and TLC (3252 mg) and conducted through column with the adsorbent Toyopearl HW-40F chromatography resin, eluted with methanol, yielding four fractions (Z1–Z4). Fraction Z2 (1594 mg) was further purified by silica gel column chromatography using a petroleum ether–acetone (PE:AT) gradient (50:1 → 1:1, *v*/*v*) and preparative HPLC purification, affording compounds **1** (29.1 mg) and **2** (11.4 mg). Subfraction A5-F (185 mg) was separated on a Toyopearl HW-40F column using methanol as the eluent to produce four fractions (F1–F4). Fraction F4 was purified by preparative chromatography to afford compound **3** (54 mg). Subfraction A5-H (325 mg) was further purified on column with Toyopearl HW-40F resin and methanol, followed by preparative chromatography of fractions H2–H5 to yield seven fractions (H1–H7). Final purification afforded compounds **4** (11 mg), **5** (3 mg) and **6** (10 mg), while the remaining fractions contained mixtures of minor constituents. Subfraction A5-I (219 mg) was passed through a column with the adsorbent HW-40F and methanol to afford five fractions (I1–I5). Fraction I4 was purified with prep-HPLC and yielded compound 7 (2 mg). Subfractions A6+A7 (18.3 g) were passed through a column with the adsorbent Sephadex-LH20, using methanol as solvent. A total of 14 subfractions (LH1–LH14) were obtained, further LH7 subfraction passed through column with silica gel, and eluent—PE:AT gradient (50:1 → 1:1, *v*/*v*) to afford compound **8** (42 mg). Compounds **9** (8 mg) was isolated from subfraction B via preparative HPLC purification.

The n-butanol fraction (58 g) was subjected to D101 macroporous resin column chromatography eluted with a gradient of ethanol:water (10:90, 30:70, 50:50, 70:30, 90:10, 100:0, *v*/*v*) to give 5 fractions (Fr.1–Fr.5). Fr.2 (1.6 g) was subjected to flash separation chromatography using ODS column LiChroprep RP-18 with gradient elution (C_2_H_5_OH:H_2_O, 10:90—100:0, *v*/*v*), to give 6 subfractions (Fr.2.1–Fr.2.6). The obtained subfractions Fr.2.2–Fr.2.4 were separated by Sephadex LH-20 column chromatography using ethanol:water (9:1, *v*/*v*) and ethanol as eluents, respectively. Subfraction Fr.2.1 yielded compound **10** (3 mg) through purification via semi-preparative HPLC (ACN:H_2_O, 19:81, *v*/*v*). From subfraction Fr.2.2, compounds **11** (12 mg) and **12** (9 mg) were obtained by semi-preparative HPLC (ACN:H_2_O, 17:73, *v*/*v*). Subfraction Fr.2.3 was purified by semi-preparative HPLC (ACN:H_2_O, 15:85, *v*/*v*) to afford compounds **13** (7 mg) and **14** (10 mg). Compound **15** (7 mg) was obtained by semi-preparative HPLC (ACN:H_2_O, 16:84, *v*/*v*) from subfraction Fr.2.4.

### 3.4. Compound Identification

The structures of the isolated monomeric compounds were determined by a combination of LC-MS spectrometry and nuclear magnetic resonance (NMR) spectroscopy. The obtained data of the spectrum were matched with those reported in the literature for known compounds to ensure the reliable identification and structural assignments of the isolated constituents.

### 3.5. Computational Section

The TDDFT ECD calculations were performed using the Gaussian 16 program [26]. The conformational searching was conducted by the Conflex 8.0 software using the MMFF force field within an energy window of 5.0 kcal/mol [27]. The conformers with the Boltzmann population above 0.1% were re-optimized at the M06-2X/6-31G(d) level with the SMD solvent model for methanol. TDDFT ECD calculations were run at the CAM-B3LYP/TZVP level with the SMD solvent model for methanol. ECD spectra were generated using the SpecDis Version 1.71 with 0.3 sigma/gamma (eV) [28,29].

### 3.6. Enzyme Activity Assays

#### 3.6.1. α-Glucosidase Inhibitory Activity Assay

The α-glucosidase (EC 3.2.1.20) inhibitory activity was evaluated using a previously published method, with slight modifications [30]. The substrate used in the assay was p-nitrophenyl-α-D-glucopyranoside (p-NPG) which was dissolved in 50 mM phosphate buffer at pH 6.8, the optimum pH for enzyme activity. Test samples were dissolved in DMSO and diluted to the desired concentrations (300, 100, 30, 10, 3, 1, 0.3, and 0.1 μM) for analysis, as was the reference inhibitor, deoxynojirimycin (DNJ).

A volume of 10 μL of the sample or DNJ solution was added to each well of a 96-well microplate, followed by 40 μL of 1.0 mM p-NPG was added, and 130 μL of phosphate buffer was added. Then, 20 μL of α-glucosidase solution (0.1 U/mL) was added to start the reaction. Immediate monitoring of the release of p-nitrophenol was performed by taking absorbance measurements at 405 nm at 37 °C. To correct for background absorbance arising from the intrinsic absorbance of the tested compounds and non-enzymatic hydrolysis of p-NPG, a no-enzyme control was prepared for each compound by replacing the α-glucosidase solution with an equal volume of phosphate buffer while keeping all other reaction components identical. The absorbance of the no-enzyme control was subtracted from that of the corresponding enzyme-containing reaction before calculating the percentage inhibition. Each sample’s inhibitory activity was presented as the concentration needed to inhibit 50% of the enzyme activity (IC_50_). The percentage of enzyme inhibition was obtained from Equation (1).

(1) $$Inhibition\;\left(\%\right)=\frac{A_{0}-A}{A_{0}}*100$$

#### 3.6.2. Assay of PTP1B Inhibitory Activity

The inhibitory activity against protein tyrosine phosphatase 1B (PTP1B, EC 3.1.3.48) was evaluated according to a previously reported procedure but with minor modifications [31]. Recombinant human PTP1B was purchased from Enzo Life Sciences Inc. (Farmingdale, New York, NY, USA). Ursolic acid (Sigma-Aldrich, St. Louis, MO, USA), which is the standard PTP1B inhibitor, was also applied in the assay. Test samples and the reference inhibitor, ursolic acid, were first dissolved in DMSO and then diluted to the appropriate concentrations (300, 100, 30, 10, 3, 1, 0.3, and 0.1 μM).

The enzyme assay was carried out in Tris-HCl buffer (25 mM, pH 7.5) with 1 mM EDTA, 2 mM 2-mercaptoethanol, and 1 mM dithiothreitol (DTT). Hydrochloric acid was used to adjust the pH of the buffer. The reactions were performed in 96-well microplates by adding 130 μL of buffer, 10 μL of test sample, 40 μL of the substrate p-nitrophenyl phosphate (pNPP, 0.8 mM) and 20 μL of the PTP1B solution (1 μg/mL). The pNPP was spectrophotometrically measured at 405 nm during incubation at 37 °C for 30 min and peaks of absorbance were measured at 10 min intervals. Non-enzymatic hydrolysis of p-NPP and any background absorbance attributable to the tested compounds were corrected by measuring a no-enzyme control reaction for each compound, in which the PTP1B solution was replaced with an equal volume of Tris-HCl buffer while all other components (sample, substrate, and buffer) were retained. The absorbance of the no-enzyme control was subtracted from that of the corresponding complete reaction prior to data analysis.

Each compound was tested for inhibitory activity, which was expressed as the half-maximal inhibitory concentration (IC_50_), defined as the concentration needed to inhibit enzyme activity by 50%.

#### 3.6.3. BNA Inhibition Assay

The anti-neuraminidase activity of the compounds was assessed using a modified version of the previously reported method [32], with adjustments to the assay conditions, including the substrate volume. The fluorogenic substrate 4-methylumbelliferyl-N-acetyl-α-D-neuraminic acid sodium salt hydrate was used and the reaction media used consisted of 50 mM sodium acetate buffer (pH 5.0).

The assay was carried out in black 96-well microplates (SPL Life Science, Pocheon, Republic of Korea). The sodium acetate buffer and the 1 mM substrate solution were each added at a volume of 160 μL per well. Later, 10 μL of the test inhibitor and 10 μL of the neuraminidase solution (0.2 U/mL) were added, thus starting the reaction. Fluorescence intensity was measured using the SpectraMax M3 Multi-Mode Microplate Reader at 37 °C with an excitation wavelength of 365 nm and an emission wavelength of 450 nm. To account for possible autofluorescence of the tested compounds and non-enzymatic hydrolysis of the fluorogenic substrate, a no-enzyme control was prepared for each compound by replacing the neuraminidase solution with an equal volume of sodium acetate buffer while maintaining all other reaction components unchanged. The fluorescence intensity of the no-enzyme control was subtracted from that of the corresponding complete reaction before calculating the percentage inhibition. Quercetin was used as the positive control and was assayed under the same conditions (300, 100, 30, 10, 3, 1, 0.3, and 0.1 μM). The inhibitory activity of tested compounds was represented by IC_50_ value, which is the concentration which causes 50% inhibition of the enzyme activity. The percentage inhibition was determined by using the Equation (1).

#### 3.6.4. DPPH Radical Scavenging Activity

The DPPH Radical Scavenging Activity (RSA) method was performed as described below. DPPH free radical scavenging activity was measured as previously reported with some modifications for 96-well microplate analysis [33]. In short, 10 μL of each sample, dissolved in DMSO, was added to 190 μL of DPPH solution prepared in ethanol. All reactions were carried out at room temperature, in the dark, for 10 min. After the incubation, the absorbance was measured at 517 nm with a SpectraMax M3 Multi-Mode Microplate Reader (Molecular Devices, LLC, San Jose, CA, USA). The radical scavenging activity was expressed as the percentage inhibition as compared with the DMSO control (Equation (1)) and antioxidant capacity was further expressed as half-maximal inhibitory concentration (IC_50_) in μM. Sample-specific blanks, consisting of the extract and solvent without DPPH, were prepared to account for the intrinsic absorbance of the tested extracts at 517 nm. The absorbance of each sample blank was subtracted from that of the corresponding extract–DPPH mixture before calculating the percentage of DPPH radical inhibition. The reference antioxidant used was Trolox, which was tested at concentrations of 300, 100, 30, 10, 3, 1, 0.3, and 0.1 μM.

#### 3.6.5. Statistical Analysis

All experiments were performed independently three times, each in triplicate. The percentage inhibition was calculated according to Equation (1), and the IC_50_ values were determined by nonlinear regression of the percentage inhibition against the logarithm of the compound concentration using a four-parameter logistic (sigmoidal dose–response with variable slope) model in GraphPad Prism (version 10, GraphPad Software, San Diego, CA, USA). The IC_50_ values are expressed as mean ± standard deviation (SD) of three independent experiments (*n* = 3). The goodness of fit (R^2^) was ≥0.98 for all fitted curves.

### 3.7. Molecular Docking Analysis

All ligands were prepared from PubChem, followed by energy minimization using the MMFF94 force field in Open Babel 3.1.1 to obtain their 3D structures for docking. The protein structures were retrieved from the RCSB Protein Data Bank (PDB). Missing atoms in the receptor were repaired using PDBFixer v2.5.0, hydrogen atoms were added at pH 7.4, and the native ligands were removed. Gasteiger charges were assigned using Open Babel 3.1.1 [34]. The grid boxes were centered on the possible native ligand binding site, with dimensions set to 60 Å × 60 Å × 60 Å and a spacing of 0.375 Å. Docking simulations were performed using AutoDock Vina 1.2.7 [35], with the exhaustiveness parameter set to 32 to ensure thorough sampling. For each ligand-protein complex, 10 conformations were generated. The conformation with the lowest binding free energy (Vina docking score) was selected for further analysis. 2D interaction diagrams and 3D visualizations were generated using LigPlot+ 2.3 and PyMOL 2.5.2 [36].

## 4. Conclusions

In this study, 15 compounds were isolated through the chromatographic separation of both dichloromethane and butanol extracts from *A. albida* Willd. Two sesquiterpenes, including the new-skeleton sesquiterpenoid Albiterpene A (**1**), together with one lignan, one fatty acid ester, and eleven phenolic compounds, were identified by spectroscopic and spectrometric analysis. Additionally, all 15 compounds were reported for the first time, expanding the knowledge of its phytochemical content and highlighting this species as a source of structurally diverse secondary metabolites. The PTP1B, α-glucosidase, and BNA inhibitory and antioxidant activities of all 15 isolates were evaluated for the first time. Absolute configuration of one new skeleton sesquiterpenoid Albiterpene A was determined by the TDDFT ECD calculation. Among all tested compounds, arteanoflavone exhibited the most potent α-glucosidase (IC_50_ = 3.2 ± 0.07 μM) and PTP1B (IC_50_ = 6.8 ± 0.14 μM) inhibition and strong DPPH radical-scavenging activity (IC_50_ = 1.5 ± 0.03 μM), outperforming the positive controls DNJ and Trolox. Notable dual inhibition of both α-glucosidase and PTP1B was also observed for caffeic acid ethyl ester and chlorogenic acid, consistent with the presence of catechol-type hydroxyl groups that enhance electron donation and enzyme binding through hydrogen bonding. Catechin and epicatechin demonstrated comparable multi-target activities, reflecting their well-known antioxidant and antidiabetic potential linked to the flavan-3-ol skeleton. The sesquiterpenoids albiterpene A and 6α-hydroxyanhydrooplopanone showed moderate α-glucosidase (IC_50_ = 24.7 ± 0.5 and 35.6 ± 0.7 μM) and PTP1B (IC_50_ = 28.0 ± 0.6 and 54.3 ± 1.1 μM) inhibition, respectively, suggesting that the presence of hydroxyl and carbonyl moieties may contribute to enzyme interaction. Collectively, the docking results indicate that chlorogenic acid achieves superior predicted binding through a high density of polar contacts and salt-bridge formation, whereas arteanoflavone relies more on π-π stacking and hydrophobic interactions, and caffeic acid ethyl ester, possessing fewer polar functional groups, exhibits comparatively weaker predicted affinity. It should be emphasized that docking scores reflect calculated binding energetics only; the stronger experimental activities of arteanoflavone suggest that factors beyond simple phenolic hydroxyl density govern target engagement in vitro. Overall, these findings highlight the phytochemical diversity and multitarget bioactivity of *A. albida* and warrant further in-depth exploration of this species.

## Figures and Tables

**Table 1:** ^1^H and ^13^C NMR data for compound **1** in CD_3_OD (600 MHz for ^1^H NMR and 150 MHz for ^13^C NMR).

No.	*δ*_H_ (*J* in Hz)	*δ*_C_	No.	*δ*_H_ (*J* in Hz)	*δ*_C_
1	4.01 (dd, *J* = 12.5, 3.4 Hz, 1H)	88.6	9	2.00 (d, *J* = 15.9 Hz, 1H) 2.38 (dd, *J* = 15.8, 1.0 Hz, 1H)	47.1
2	1.86 (m, 1H) 1.99 (m, 1H)	22.1	10	–	46.5
3	1.63 (ddd, *J* = 14.4, 12.6, 4.7 Hz, 1H) 1.93 (m, 1H)	39.9	11	2.32 (m, 1H)	32.7
4	–	72.4	12	1.02 (d, *J* = 6.8 Hz, 3H)	23.2
5	2.01 (d, *J* = 6.8 Hz, 1H)	57.5	13	1.01 (d, *J* = 6.8 Hz, 3H)	23.1
6	5.66 (ddd, *J* = 15.4, 9.5, 1.2 Hz)	124.2	14	1.17 (d, *J* = 1.2 Hz, 3H)	14.9
7	5.52 (dd, *J* = 15.4, 6.7 Hz)	142.9	15	1.10 (s, 3H)	30.7
8	–	179.9			

**Table 2:** Activity profile of isolated compounds from *A. albida* Willd.

No.	Compounds	α-Glucosidase	PTP1B	BNA	DPPH
		IC_50_ (μM)	IC_50_ (μM)	IC_50_ (μM)	IC_50_ (μM)
**1**	Albiterpene A	24.7 ± 0.5	28.0 ± 0.6	25.5 ± 0.5	12.4 ± 0.2
**2**	6-α hydroxy anhydrooplopanone	35.6 ± 0.7	54.3 ± 1.1	29.8 ± 0.6	22.4 ± 0.5
**3**	(−)-(7R,8S,7′E)-4-Hydroxy-3,5′-dimethoxy-7,4′-epoxy-8,3′-neolign-7′-ene-9,9′-diol 9′-ethyl ether	27.2 ± 0.5	56.1 ± 1.2	35.4 ± 0.7	7.0 ± 0.16
**4**	Arteanoflavone	3.2 ± 0.07	6.8 ± 0.14	8.3 ± 0.17	1.5 ± 0.03
**5**	5,7,4′-trihydroxy-6,3′,5′-trimethoxyflavone	42.4 ± 0.9	58.8 ± 1.2	33.4 ± 0.7	12.0 ± 0.3
**6**	5,3′-dihydroxy-6,7,4′,5′-trimethoxyflavanone	55.4 ± 1.2	87.4 ± 1.8	41.6 ± 0.8	18.6 ± 0.4
**7**	Homoeriodictyol	64.7 ± 1.3	78.3 ± 1.6	39.8 ± 0.8	13.6 ± 0.3
**8**	Caffeic acid ethyl ester	8.2 ± 0.1	24.2 ± 0.5	14.2 ± 0.3	3.6 ± 0.08
**9**	Ethyl tridecanoate	NA	NA	NA	NA
**10**	Chlorogenic acid	17.3 ± 0.3	34.2 ± 0.7	21.8 ± 0.4	6.3 ± 0.14
**11**	(+)-catechin	23.7 ± 0.5	45.6 ± 1.0	18.2 ± 0.4	2.5 ± 0.05
**12**	Epicatechin	18.2 ± 0.4	17.3 ± 0.4	16.9 ± 0.3	1.3 ± 0.03
**13**	Nepitrin	76.1 ± 1.6	85.7 ± 1.8	43.4 ± 0.9	12.6 ± 0.3
**14**	Isovitexin	46.1 ± 0.8	105.9 ± 2.2	52.2 ± 1.1	21.5 ± 0.5
**15**	Vitexin-2′′-O-β-D-glucopyranoside	62.2 ±1.3	124.2 ± 2.6	58.4 ± 1.2	24.3 ± 0.6
	DNJ *	42.5 ± 0.9	-	-	-
	Ursolic acid		15.5 ± 0.3		
	Quercetin			20.2 ± 0.8	
	Trolox	-	-	-	12.8 ± 0.3

* Deoxynojirimycin (DNJ) is used as a positive control for α-glucosidase. Ursolic acid is used as a positive control for PTP1B. Quercetin is used as a positive control for BNA. Trolox is used as a positive control for DPPH. NA means no activity. All experiments were performed in three independent experiments, each in triplicate. The IC_50_ values are expressed as mean ± standard deviation (SD).

**Table 3:** Vina docking scores of test compounds against PTP1B (1NNY) and human α-glucosidase (2QM*J*).

Compounds	Vina Docking Score (kcal/mol) for 1NNY	Vina Docking Score (kcal/mol) for 2QM*J*
Arteanoflavone	−6.46	−6.80
Chlorogenic acid	−7.56	−7.70
Caffeic acid ethyl ester	−5.73	−6.17

## Data Availability

Data is contained within the article or Supplementary Materials.

## Supplementary Materials

The following supporting information can be downloaded at: https://www.mdpi.com/article/10.3390/molecules31213975/s1. Figure S1. ^1^H NMR spectrum of compound **1** (600 MHz, CD_3_OD), Figure S2. ^13^C NMR spectrum of compound **1** (150 MHz, CD_3_OD), Figure S3. HSQC spectrum of compound **1** (600 MHz, CD_3_OD), Figure S4. HMBC spectrum of compound **1** (600 MHz, CD_3_OD), Figure S5. ^1^H-^1^H COSY spectrum of compound **1** (600 MHz, CD_3_OD), Figure S6. NOESY spectrum of compound **1** (600 MHz, CD_3_OD), Figure S7. Mass spectra of compound **1**, Figure S8. HRMS EIC of compound **1**, Figure S9. HRMS—[M+H-H_2_O] of compound **1**, Figure S10. HRMS—[M+Na] of compound **1**, Figure S11. ^1^H NMR spectrum of compound **2** (600 MHz, CD_3_OD), Figure S12. ^13^C NMR spectrum of compound **2** (150 MHz, CD_3_OD), Figure S13. HSQC spectrum of compound **2** (600 MHz, CD_3_OD), Figure S14. HMBC spectrum of compound **2** (600 MHz, CD_3_OD), Figure S15. ^1^H-^1^H COSY spectrum of compound **2** (600 MHz, CD_3_OD), Figure S16. NOESY spectrum of compound **2** (600 MHz, CD_3_OD), Figure S17. ^1^H NMR spectrum of compound **3** (600 MHz, CD_3_OD), Figure S18. ^13^C NMR spectrum of compound **3** (150 MHz, CD_3_OD), Figure S19. ^1^H NMR spectrum of compound **4** (600 MHz, CD_3_OD), Figure S20. ^13^C NMR spectrum of compound **4** (150 MHz, CD_3_OD), Figure S21. ^1^H NMR spectrum of compound **5** (600 MHz, CD_3_OD), Figure S22. ^13^C NMR spectrum of compound **5** (150 MHz, CD_3_OD), Figure S23. ^1^H NMR spectrum of compound **6** (600 MHz, CD_3_OD), Figure S24. ^13^C NMR spectrum of compound **6** (150 MHz, CD_3_OD), Figure S25. ^1^H NMR spectrum of compound **7** (600 MHz, CD_3_OD), Figure S26 ^13^C NMR spectrum of compound **7** (150 MHz, CD_3_OD), Figure S27. ^1^H NMR spectrum of compound **8** (600 MHz, CD_3_OD), Figure S28. ^13^C NMR spectrum of compound **8** (150 MHz, CD_3_OD), Figure S29. ^1^H NMR spectrum of compound **9** (600 MHz, CD_3_OD), Figure S30. ^13^C NMR spectrum of compound **9** (150 MHz, CD_3_OD), Figure S31. ^1^H NMR spectrum of compound **10** (600 MHz, CD_3_OD), Figure S32. ^13^C NMR spectrum of compound **10** (150 MHz, CD_3_OD), Figure S33. ^1^H NMR spectrum of compound **11** (600 MHz, CD_3_OD), Figure S34. ^13^C NMR spectrum of compound **11** (150 MHz, CD_3_OD), Figure S35. ^1^H NMR spectrum of compound **12** (600 MHz, CD_3_OD), Figure S36. ^13^C NMR spectrum of compound **12** (150 MHz, CD_3_OD), Figure S37. ^1^H NMR spectrum of compound **13** (600 MHz, CD_3_OD), Figure S38. ^13^C NMR spectrum of compound **13** (150 MHz, CD_3_OD), Figure S39. ^1^H NMR spectrum of compound **14** (600 MHz, CD_3_OD), Figure S40. ^13^C NMR spectrum of compound **14** (150 MHz, CD_3_OD), Figure S41. ^1^H NMR spectrum of compound **15** (600 MHz, DMSO-d_6_), Figure S42. ^13^C NMR spectrum of compound **15** (150 MHz, DMSO-d_6_). Figure S43. Molecular docking poses of test compounds with (A–C) PTP1B (1NNY) and (D–F) human α glucosidase (2QMJ). (A,D) Arteanoflavone; (B,E) Chlorogenic acid; (C,F) Caffeic acid ethyl ester. Hydrogen bonds are depicted as dashed yellow lines; π-π stacking and cation-π interactions are indicated by green arrows. Key interacting residues are labeled. Table S1. Summary of conformers selected for re-optimization and their relative free energies and Boltzmann populations.

## Abbreviations

The following abbreviations are used in this manuscript:

HRESIMS	High-Resolution Electrospray Ionization Mass Spectrometry
NMR	Nuclear Magnetic Resonance
ECD	Electronic Circular Dichroism
TDDFT	Time-Dependent Density Functional Theory
PTP1B	Protein tyrosine phosphatase 1B
BNA	Bacterial neuraminidase
DPPH	2,2-Diphenyl-1-picrylhydrazyl
DNJ	1-Deoxynojirimycin
MeOH	Methanol
HSQC	Heteronuclear Single Quantum Coherence
HMBC	Heteronuclear Multiple Bond Correlation
COESY	Correlated Spectroscopy
NOESY	Nuclear Overhauser Effect Spectroscopy
DMSO	Dimethyl Sulfoxide
DCM	Dichloromethane
LC-MS	Liquid Chromatography–Mass Spectrometry
TLC	Thin-Layer Chromatography
HPLC	High-Performance Liquid Chromatography
ODS	Octadecylsilane
ACN	Acetonitrile
EDTA	Ethylenediaminetetraacetic Acid
DTT	Dithiothreitol
RSA	Radical Scavenging Activity
