# Supplementary material for: Phytochemical Profiles of Artemisia albida Willd.: Potential for PTP1B, α-Glucosidase, BNA, DPPH and Molecular Docking Analysis
Source: Molecules. 2026 Nov 4;31(21):3975. doi: 10.3390/molecules31213975 (PMC13765131; doi:10.3390/molecules31213975)
Supplement: Supplementary file 1 [file molecules-31-03975-s001.zip › molecules-4646512-supplementary.pdf]

Supporting information

1. Spectral data of compound **1**

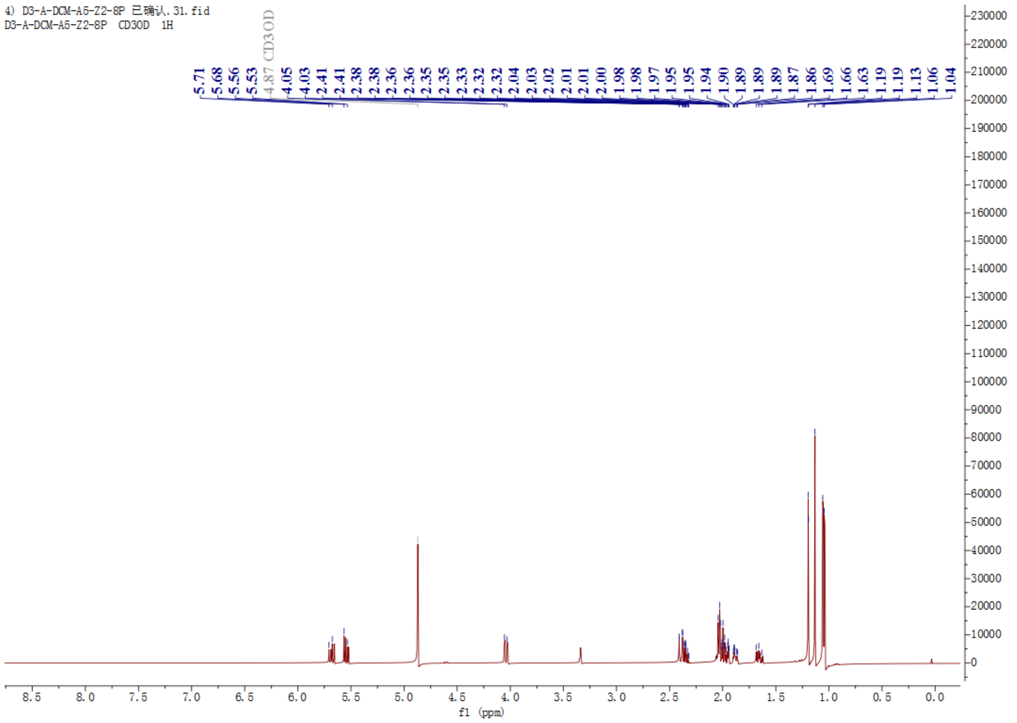

Figure S1. ^{1}H NMR spectrum of compound **1** (600 MHz, CD_3OD)

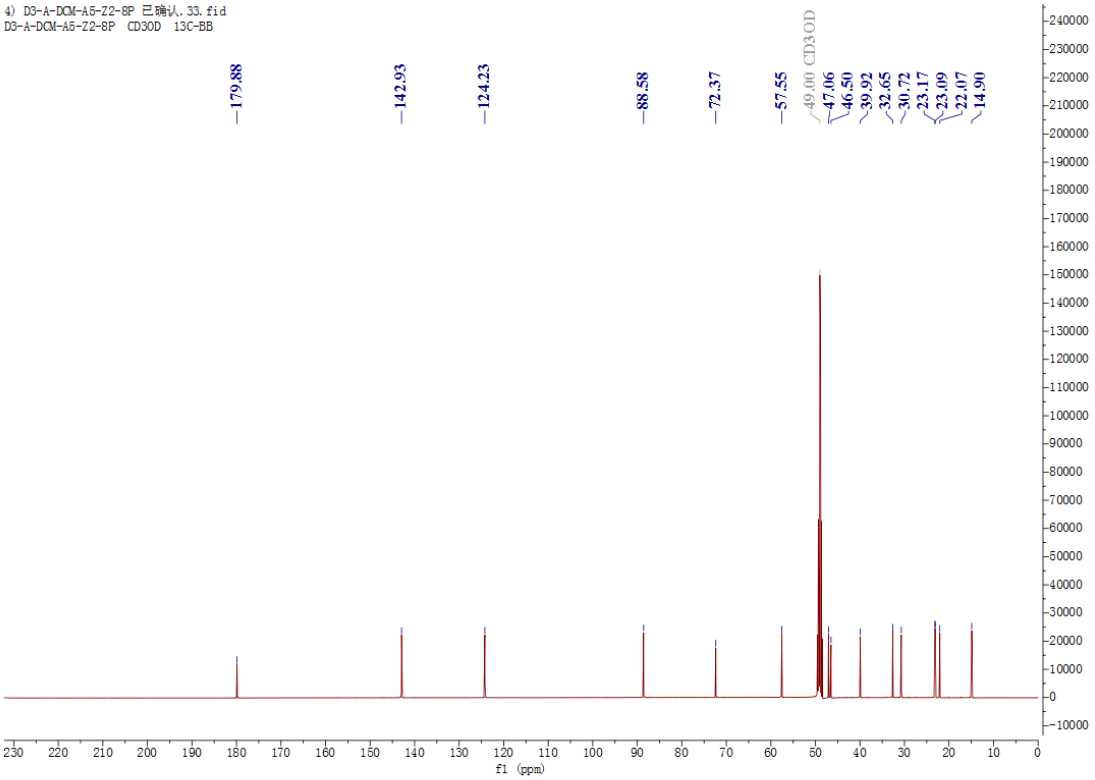

Figure S2. ^{13}C NMR spectrum of compound **1** (150 MHz, CD_3OD)

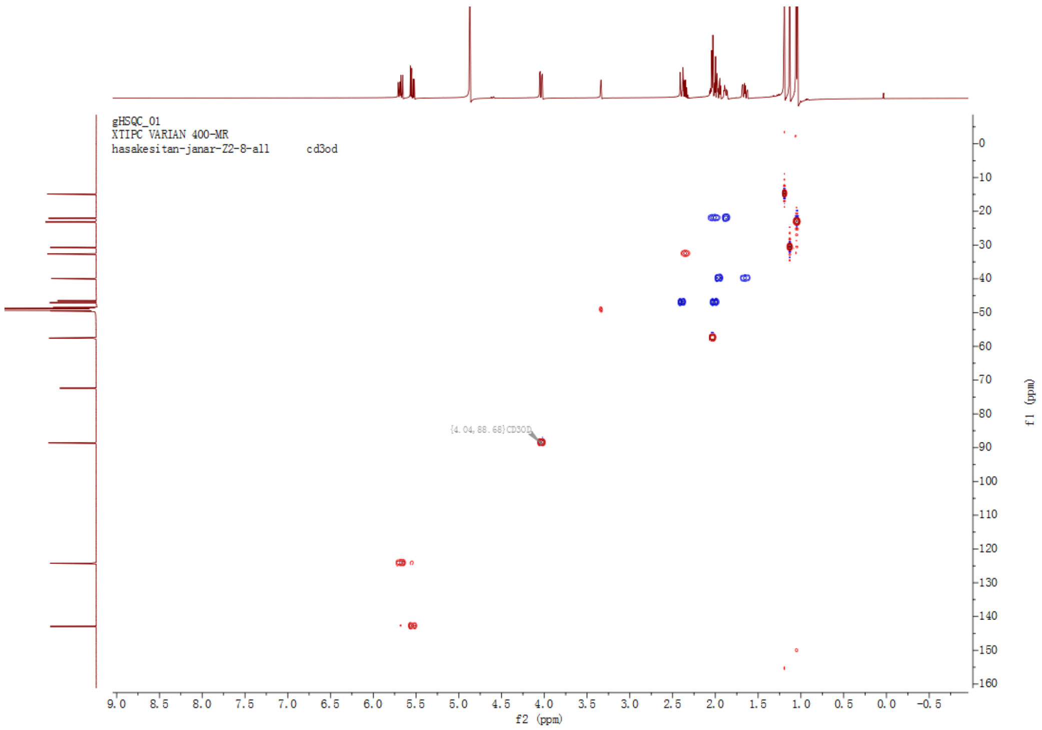

Figure S3. HSQC spectrum of compound **1** (600 MHz, CD_3OD)

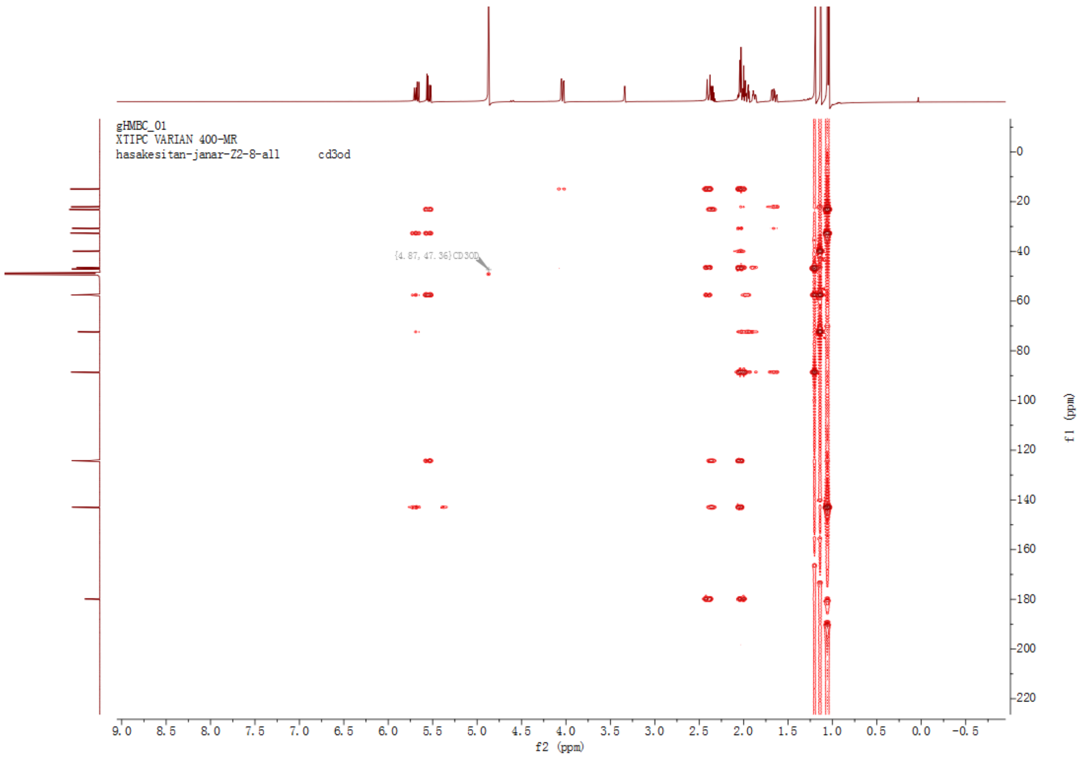

Figure S4. HMBC spectrum of compound **1** (600 MHz, CD_3OD)

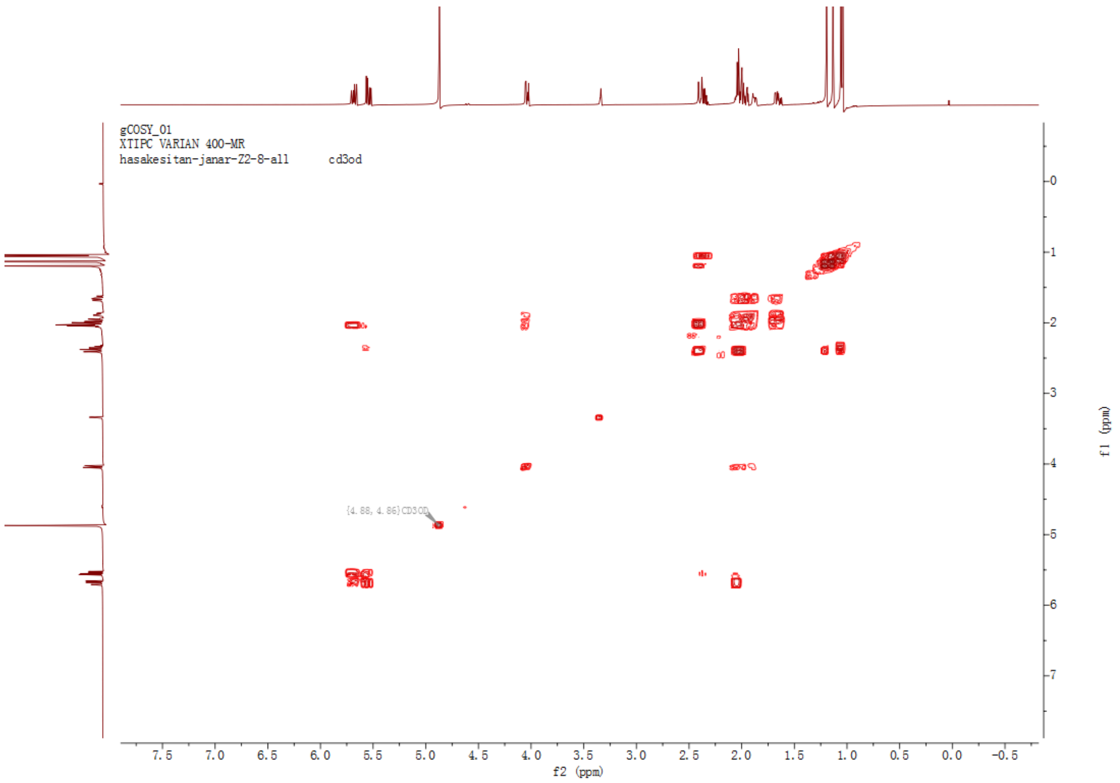

Figure S5. ^{1}H-^{1}H COSY spectrum of compound **1** (600 MHz, CD_3OD)

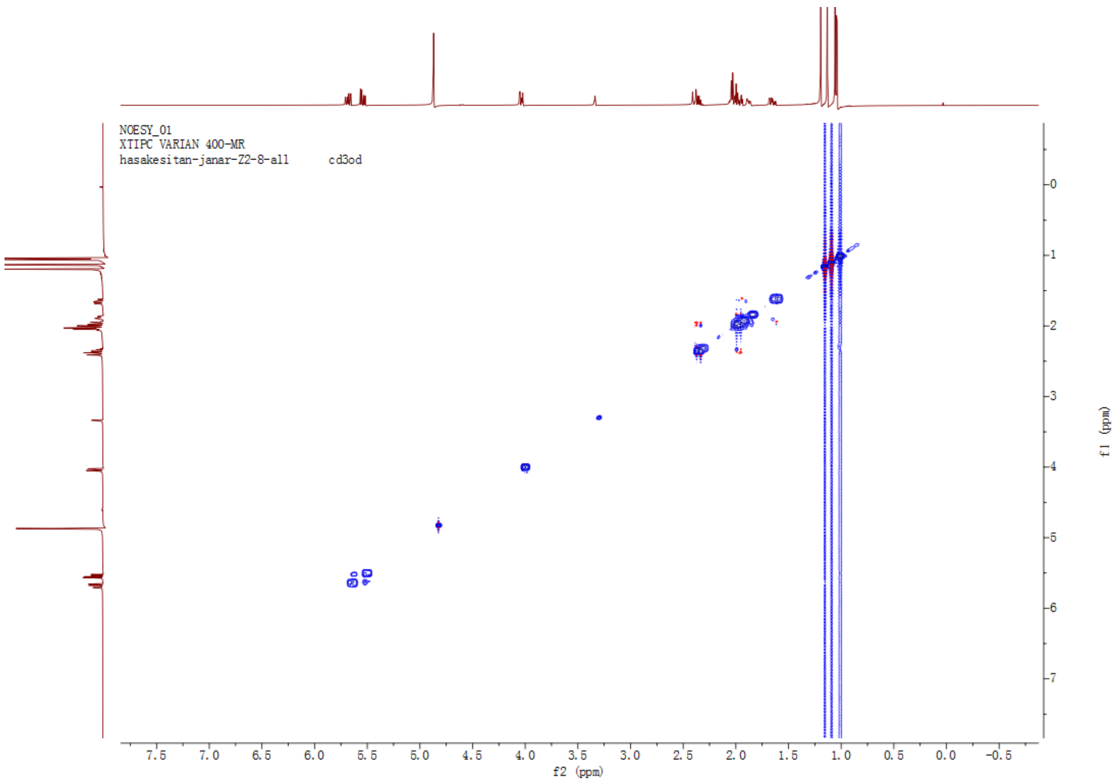

Figure S6. NOESY spectrum of compound **1** (600 MHz, CD_3OD)

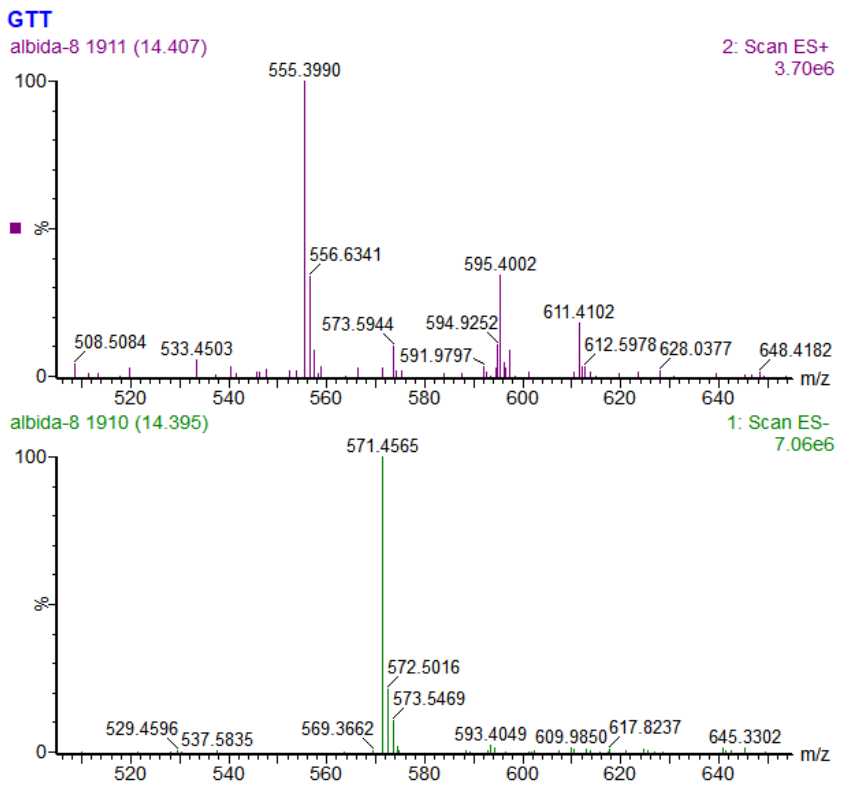

Figure S7. Mass spectra of compound **1**

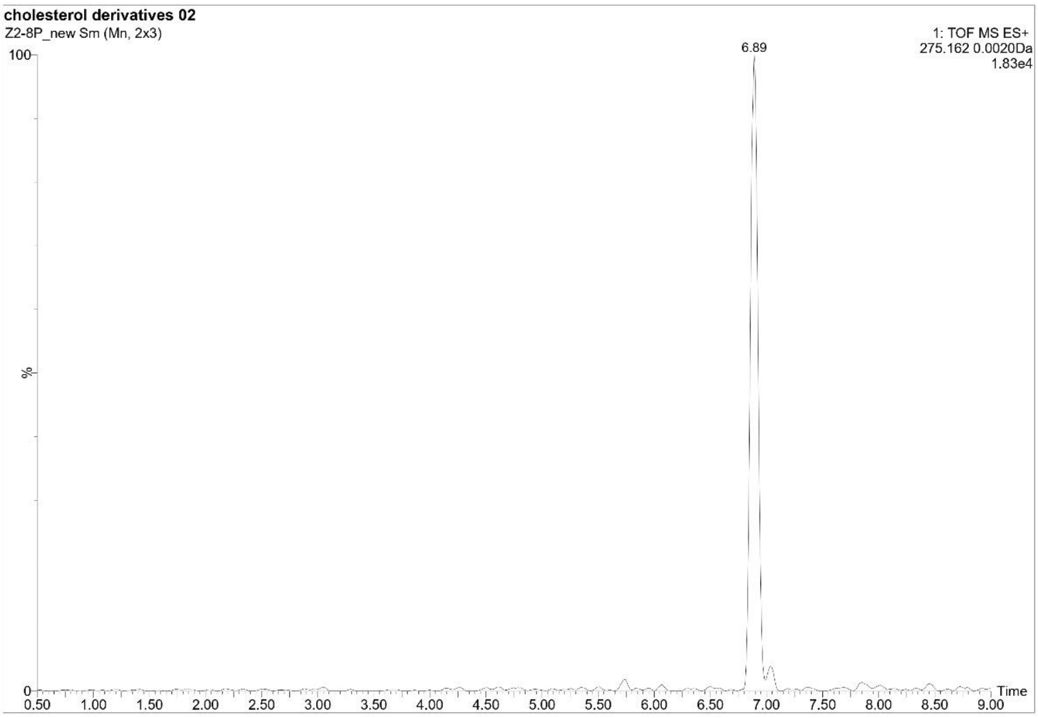

Figure S8. HRMS EIC of compound **1**

Elemental Composition Report

Tolerance = 5.0 PPM / DBE: min = -1.5, max = 50.0
Element prediction: Off
Number of isotope peaks used for i-FIT = 3

Monoisotopic Mass, Even Electron Ions
130 formula(e) evaluated with 1 results within limits (up to 5 closest results for each mass)
Elements Used:
C: 0-50 H: 0-200 N: 0-1 O: 0-20 Na: 0-1
Janar
Z2-8P_new 927 (6.904)

1: TOF MS ES+
3.14e+005

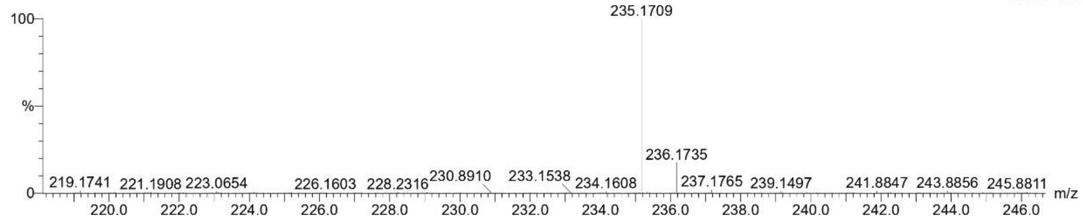

Minimum:	80.00				-1.5				
Maximum:	100.00		2.0	5.0	50.0				

Mass	RA	Calc. Mass	mDa	PPM	DBE	i-FIT	Norm	Conf(%)	Formula
235.1709	100.00	235.1698	1.1	4.7	4.5	711.4	n/a	n/a	C15 H23 O2

Figure S9. HRMS – [$M+H-H_2O$] of compound **1**

Elemental Composition Report

Page 1

Tolerance = 5.0 PPM / DBE: min = -1.5, max = 50.0
Element prediction: Off
Number of isotope peaks used for i-FIT = 3

Monoisotopic Mass, Even Electron Ions
90 formula(e) evaluated with 1 results within limits (up to 5 closest results for each mass)
Elements Used:
C: 0-50 H: 0-200 O: 0-20 Na: 0-1
Janar
Z2-8P_new 927 (6.904)

1: TOF MS ES+
1.97e+004

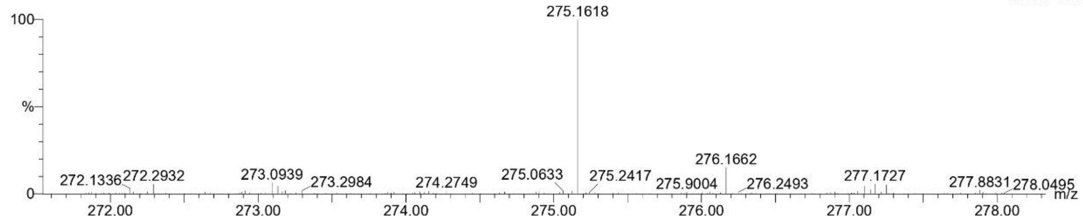

Minimum:	80.00				-1.5				
Maximum:	100.00		2.0	5.0	50.0				

Mass	RA	Calc. Mass	mDa	PPM	DBE	i-FIT	Norm	Conf(%)	Formula
275.1618	100.00	275.1623	-0.5	-1.8	3.5	474.3	n/a	n/a	C15 H24 O3 Na

Figure S10. HRMS – [M+Na] of compound **1**

2. Spectral data of compound **2**

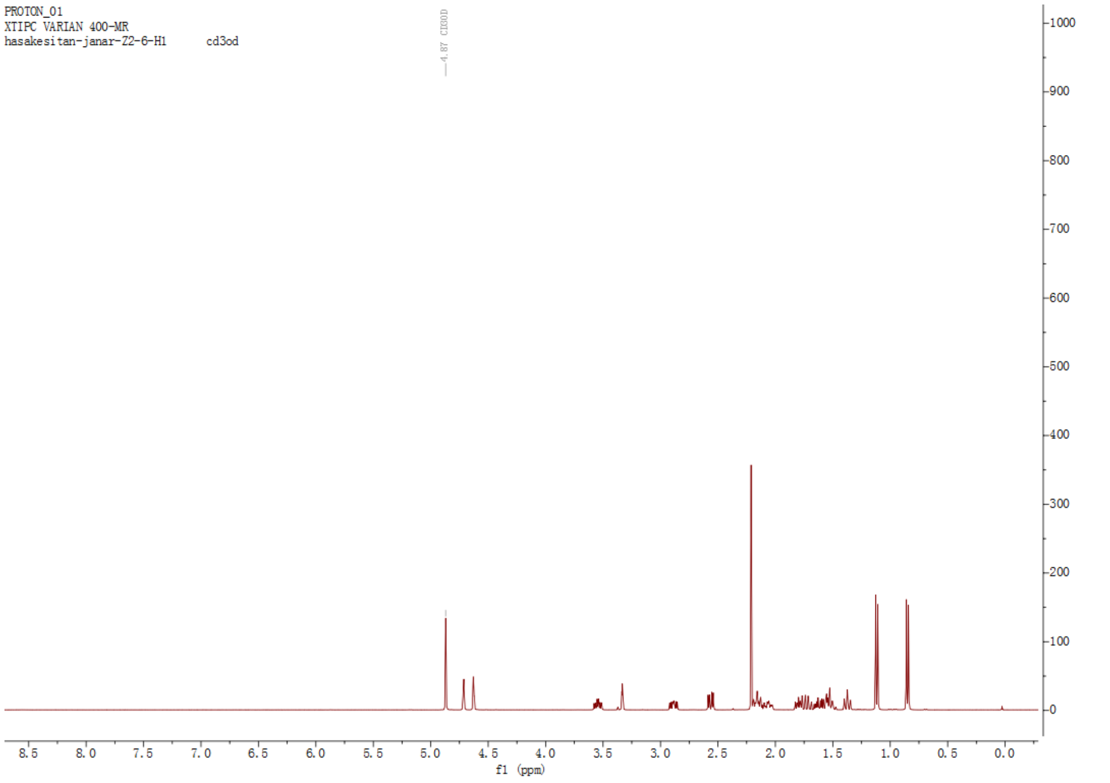

Figure S11. ^{1}H NMR spectrum of compound **2** (600 MHz, CD_3OD)

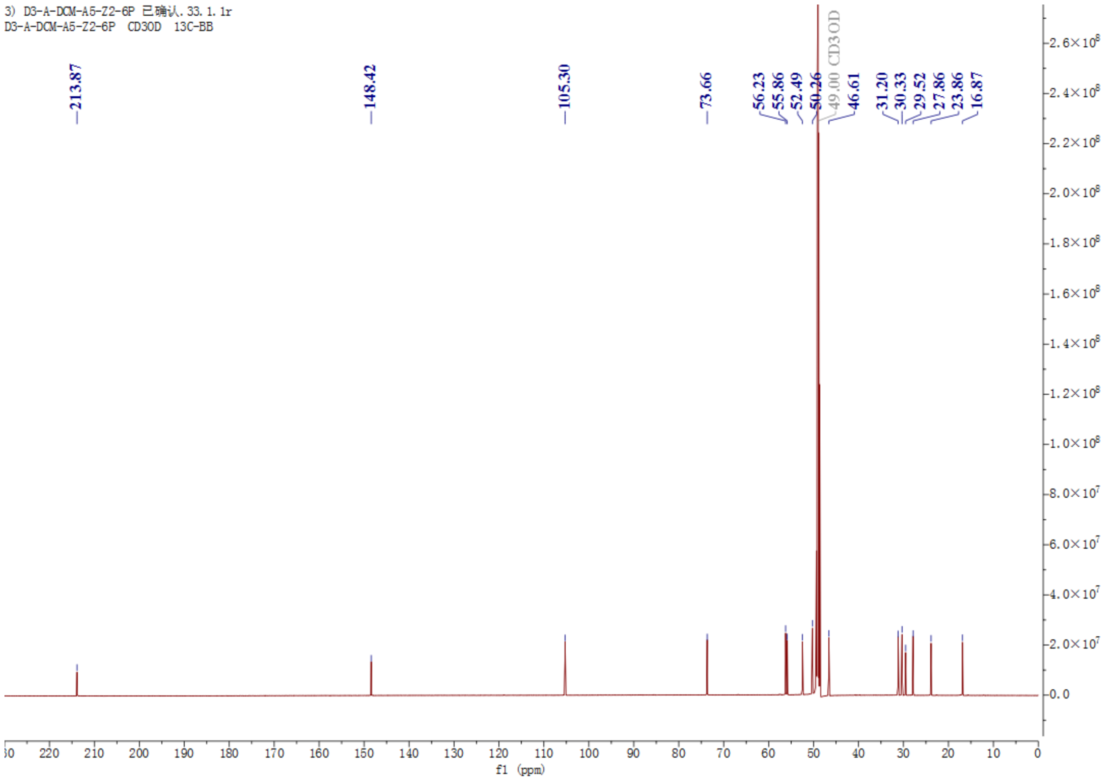

Figure S12. ^{13}C NMR spectrum of compound **2** (150 MHz, CD_3OD)

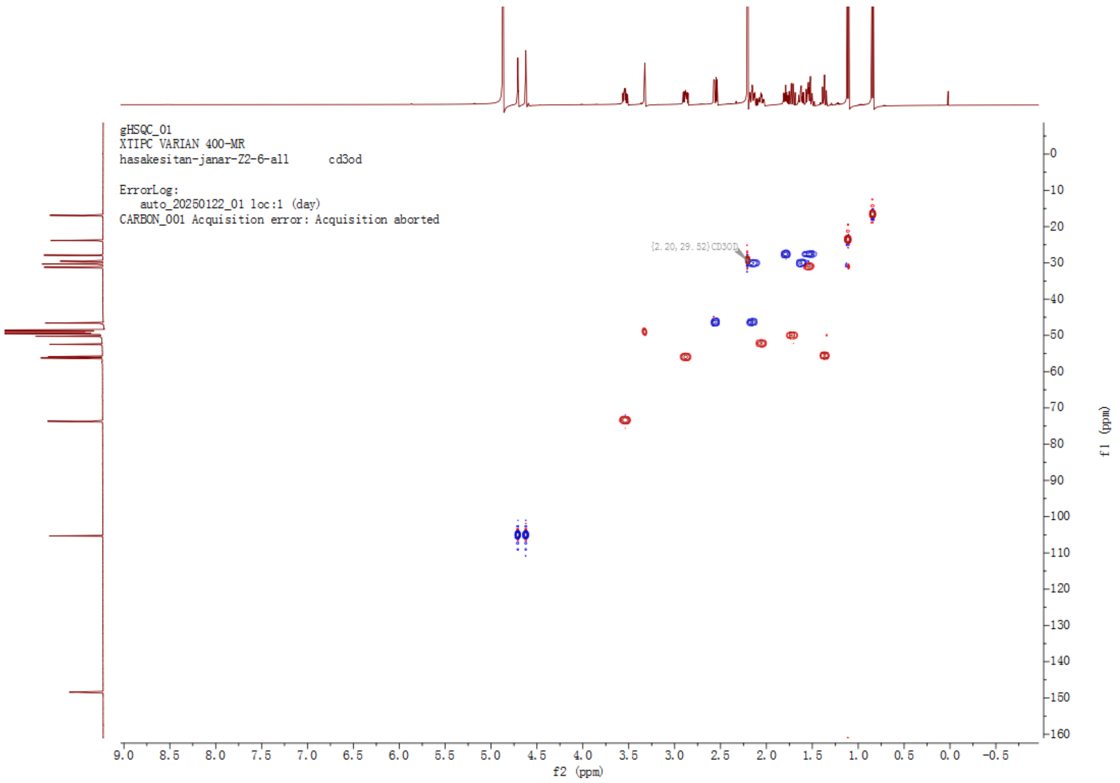

Figure S13. HSQC spectrum of compound **2** (600 MHz, CD_3OD)

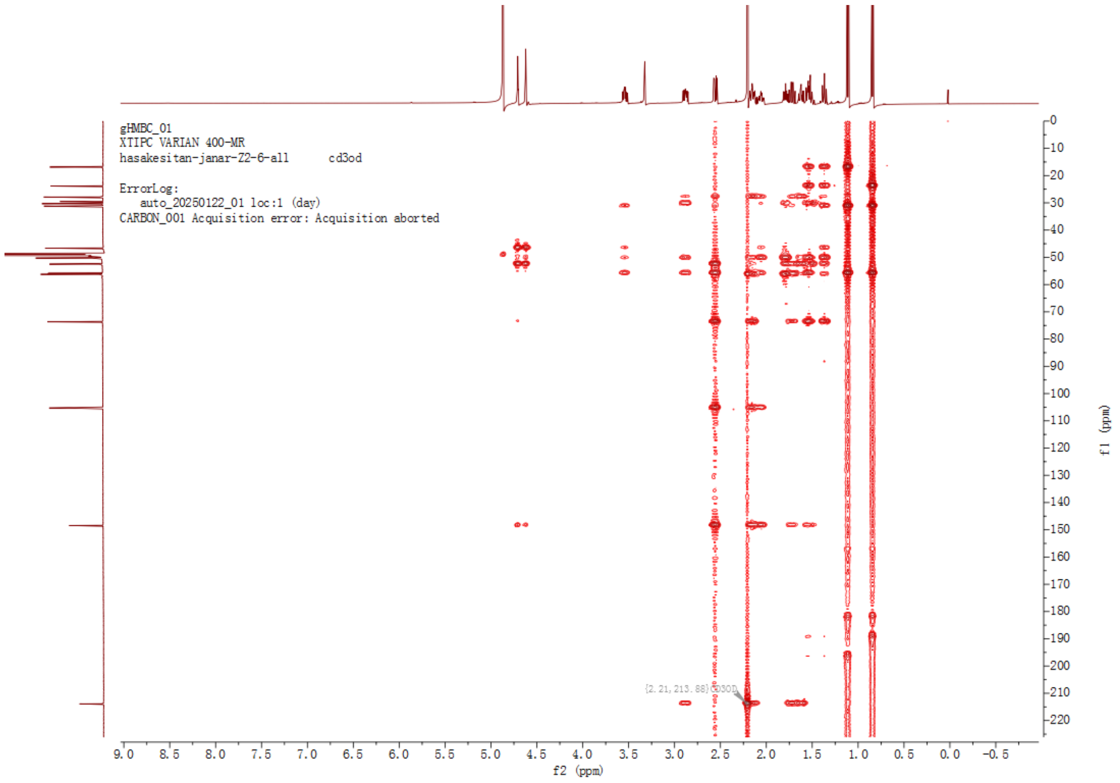

Figure S14. HMBC spectrum of compound **2** (600 MHz, CD_3OD)

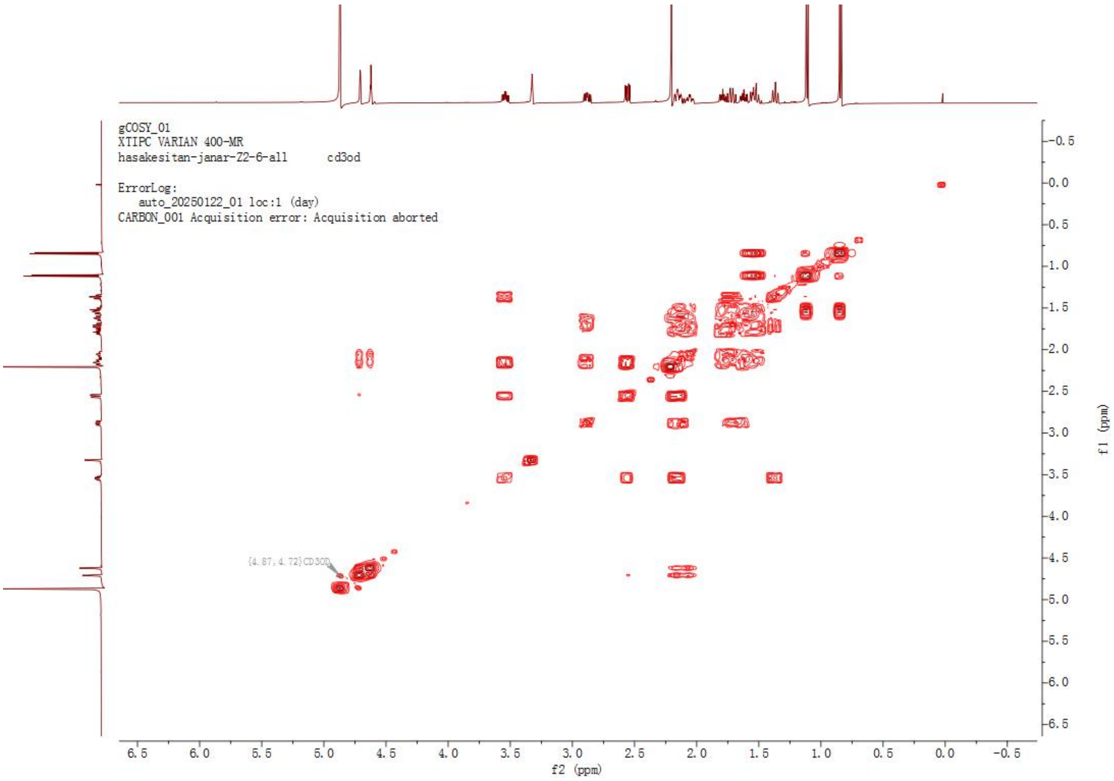

Figure S15. ^1H-^1H COSY spectrum of compound **2** (600 MHz, CD_3OD)

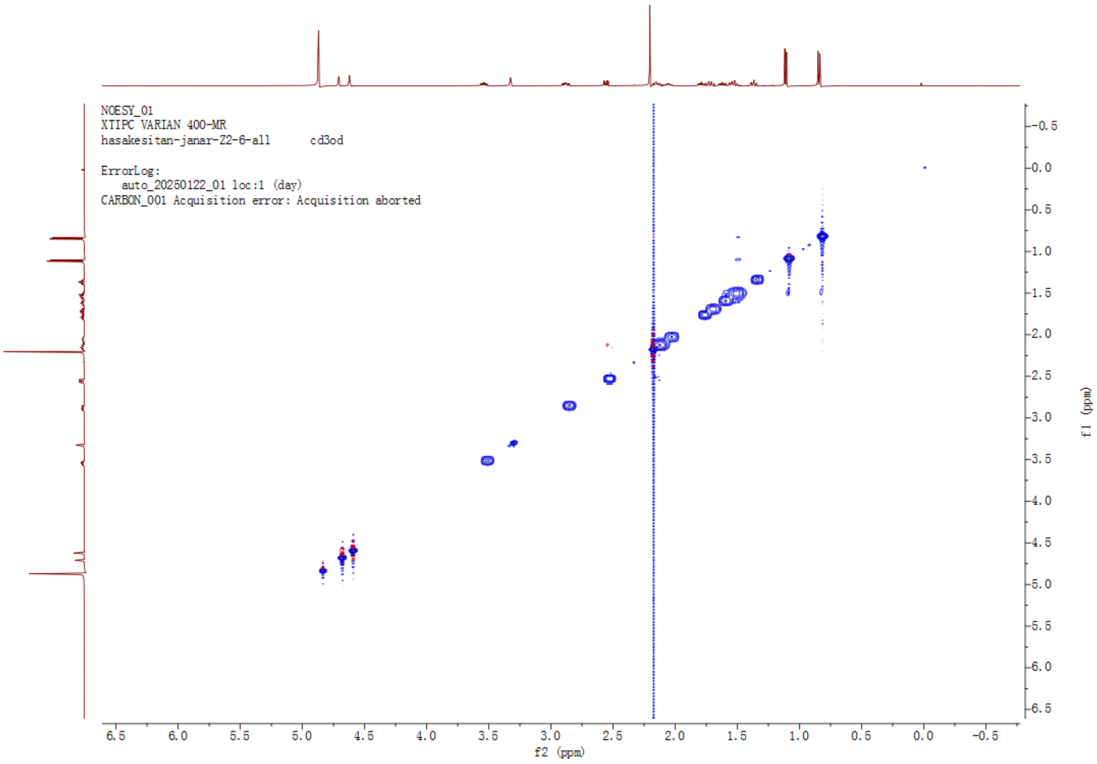

Figure S16. NOESY spectrum of compound **2** (600 MHz, CD_3OD)

3. Spectral data of compound **3**

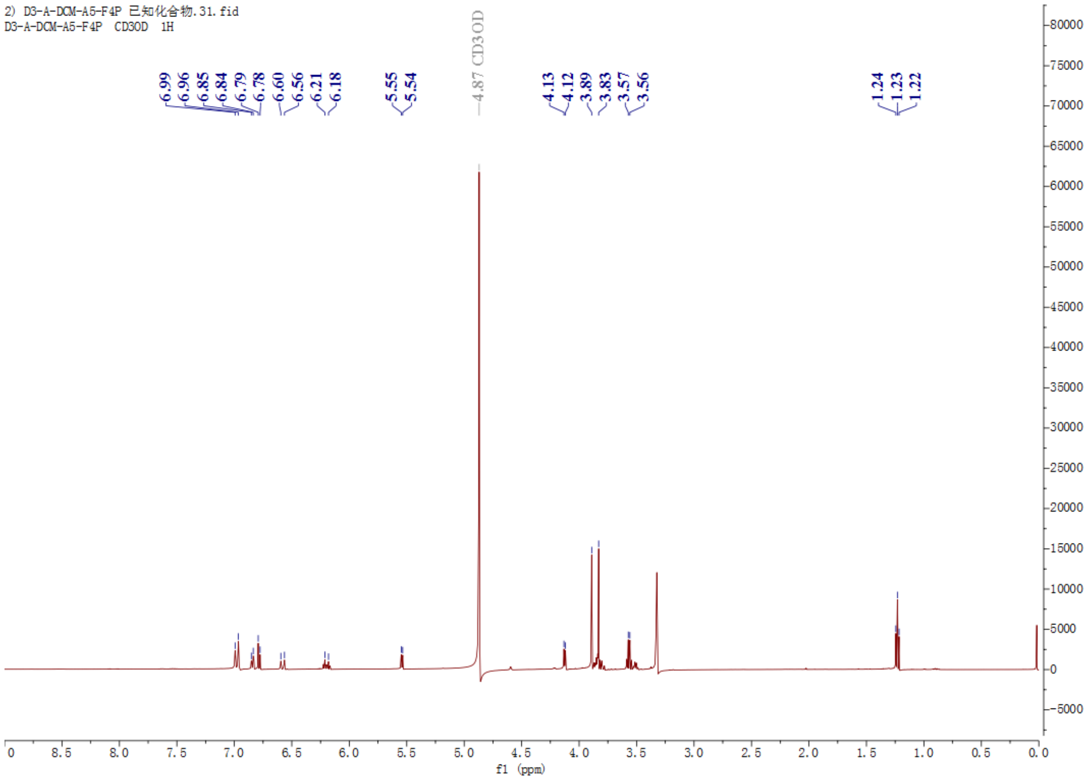

Figure S17. ^{1}H NMR spectrum of compound **3** (600 MHz, CD_3OD)

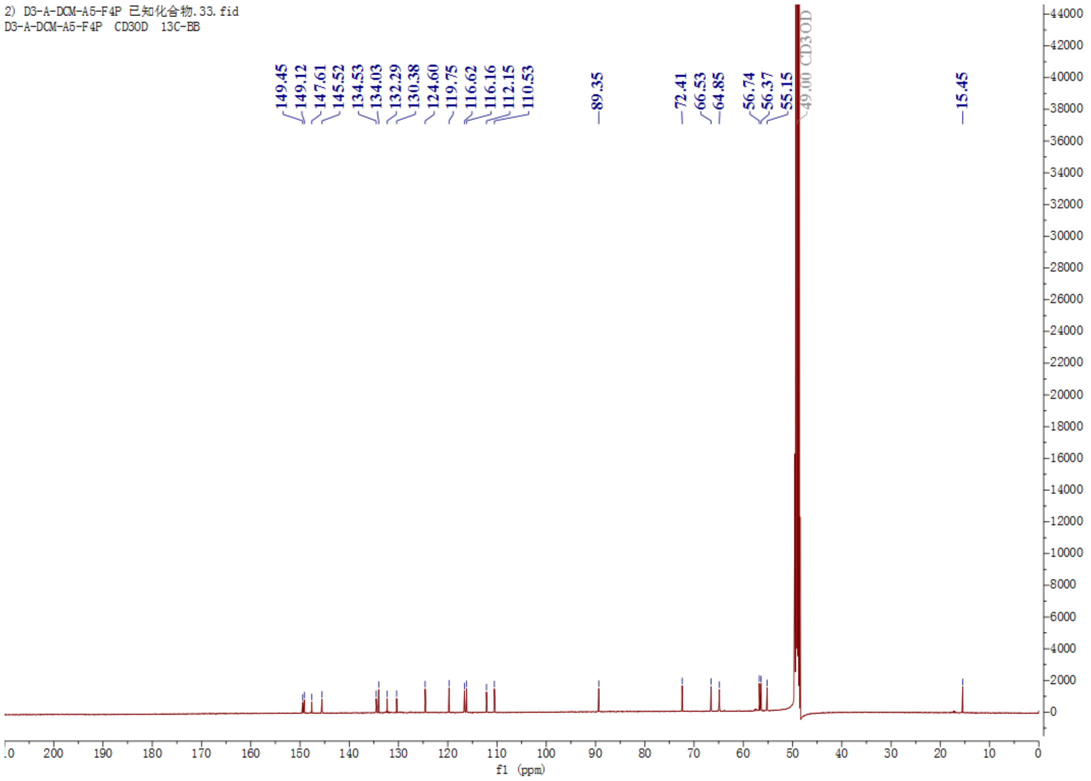

Figure S18. ^{13}C NMR spectrum of compound **3** (150 MHz, CD_3OD)

4. Spectral data of compound **4**

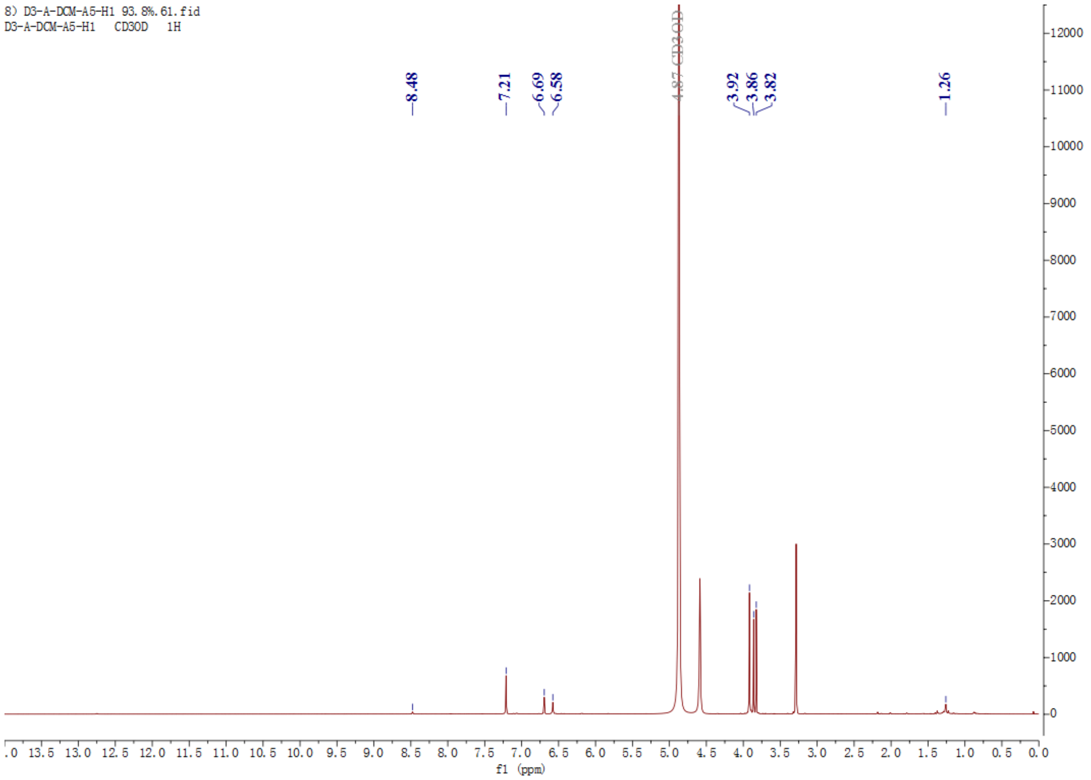

Figure S19. ^{1}H NMR spectrum of compound **4** (600 MHz, CD_3OD)

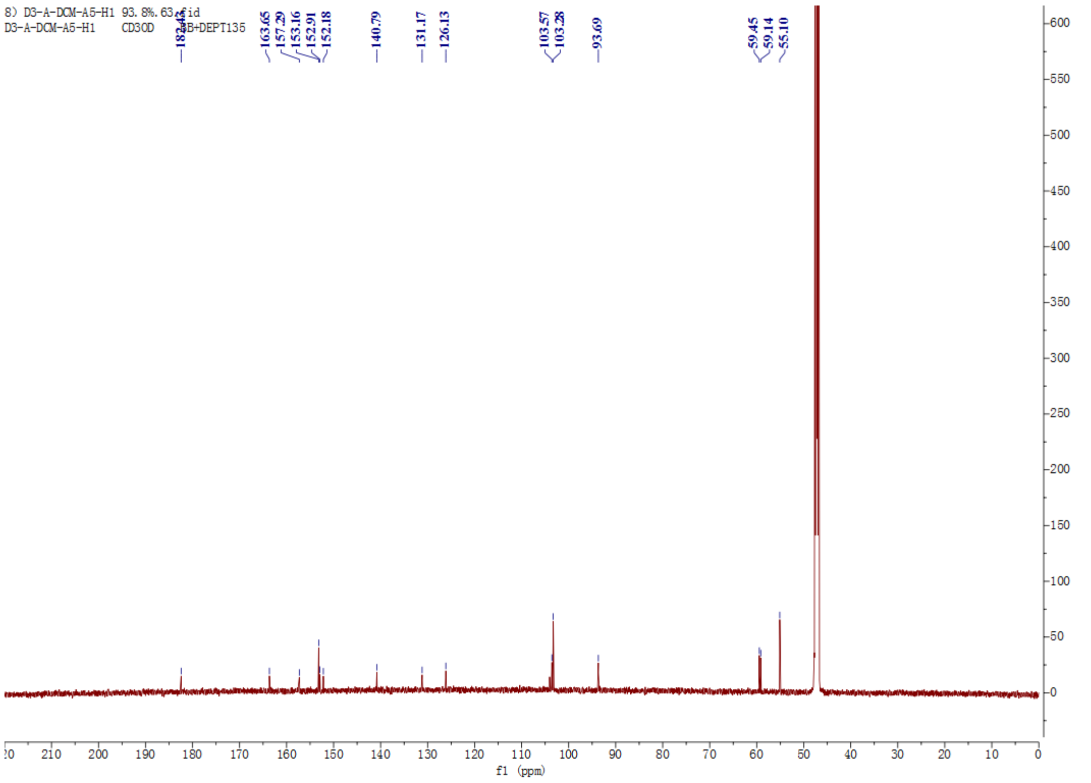

Figure S20. ^{13}C NMR spectrum of compound **4** (150 MHz, CD_3OD)

5. Spectral data of compound **5**

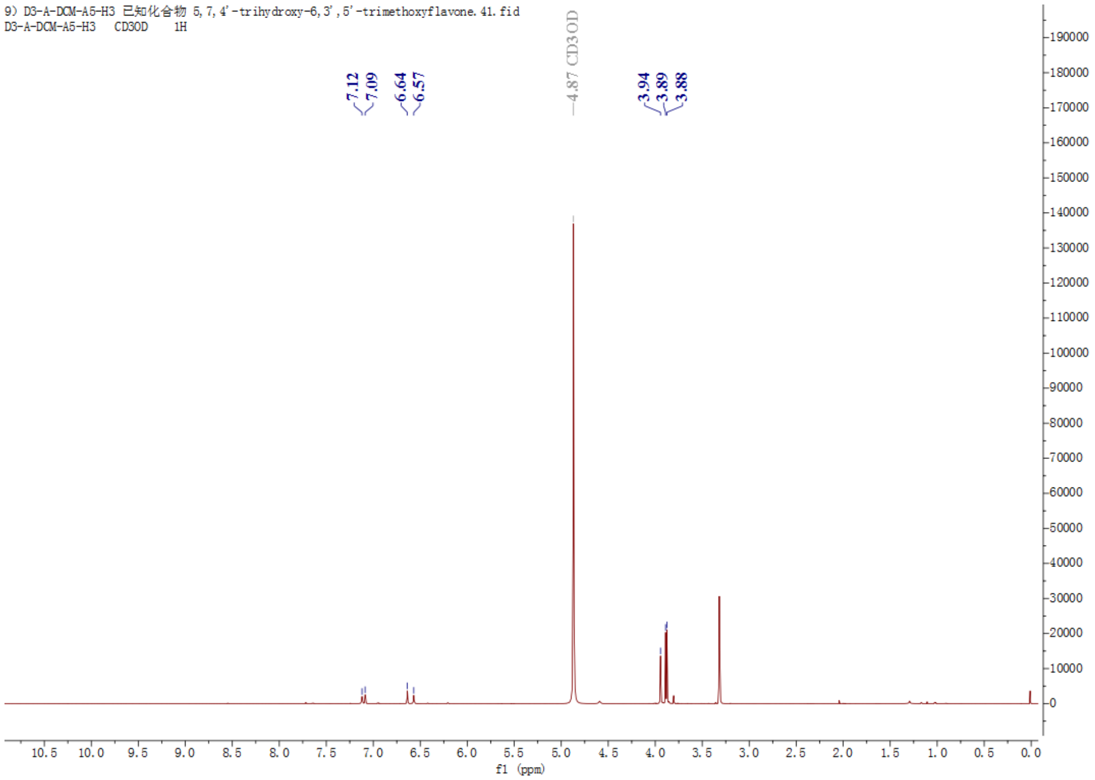

Figure S21. ^{1}H NMR spectrum of compound **5** (600 MHz, CD_3OD)

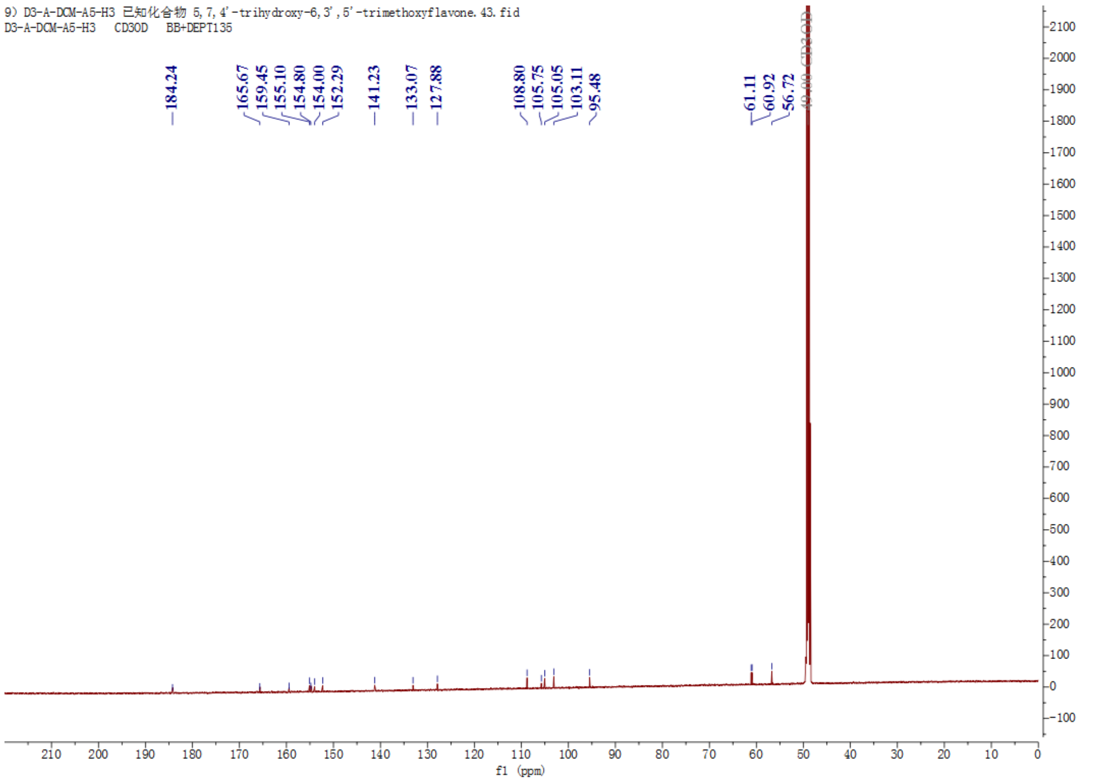

Figure S22. ^{13}C NMR spectrum of compound **5** (150 MHz, CD_3OD)

6. Spectral data of compound **6**

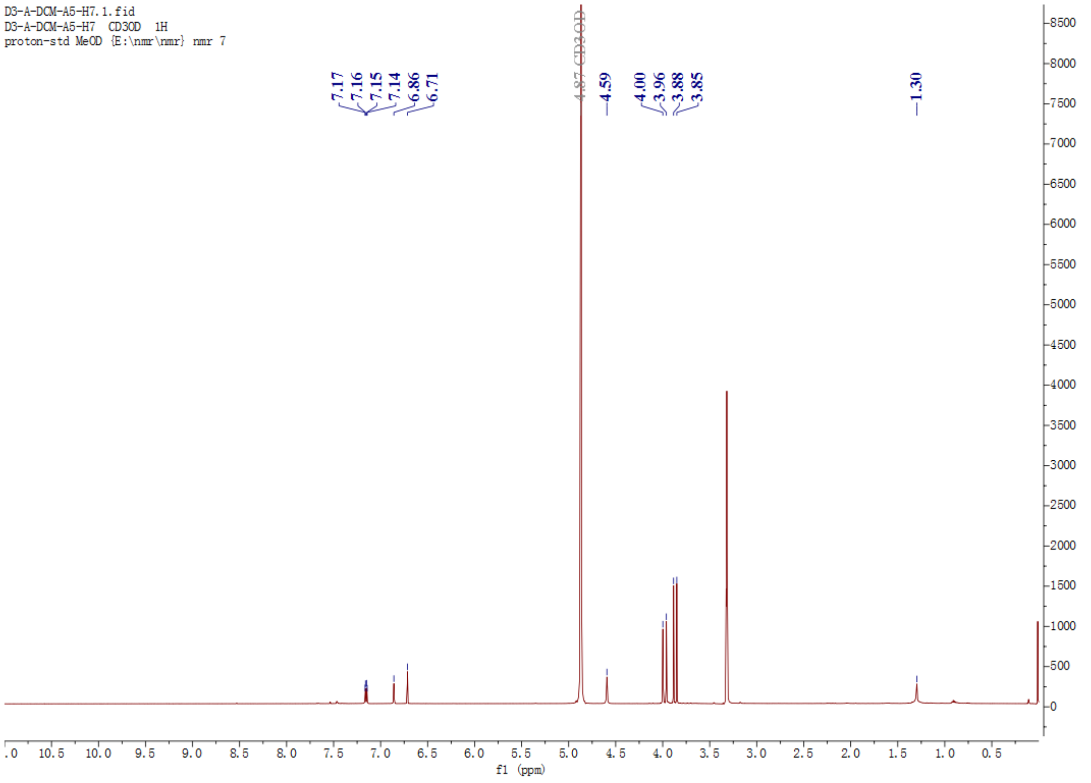

Figure S23. ^{1}H NMR spectrum of compound **6** (600 MHz, CD_3OD)

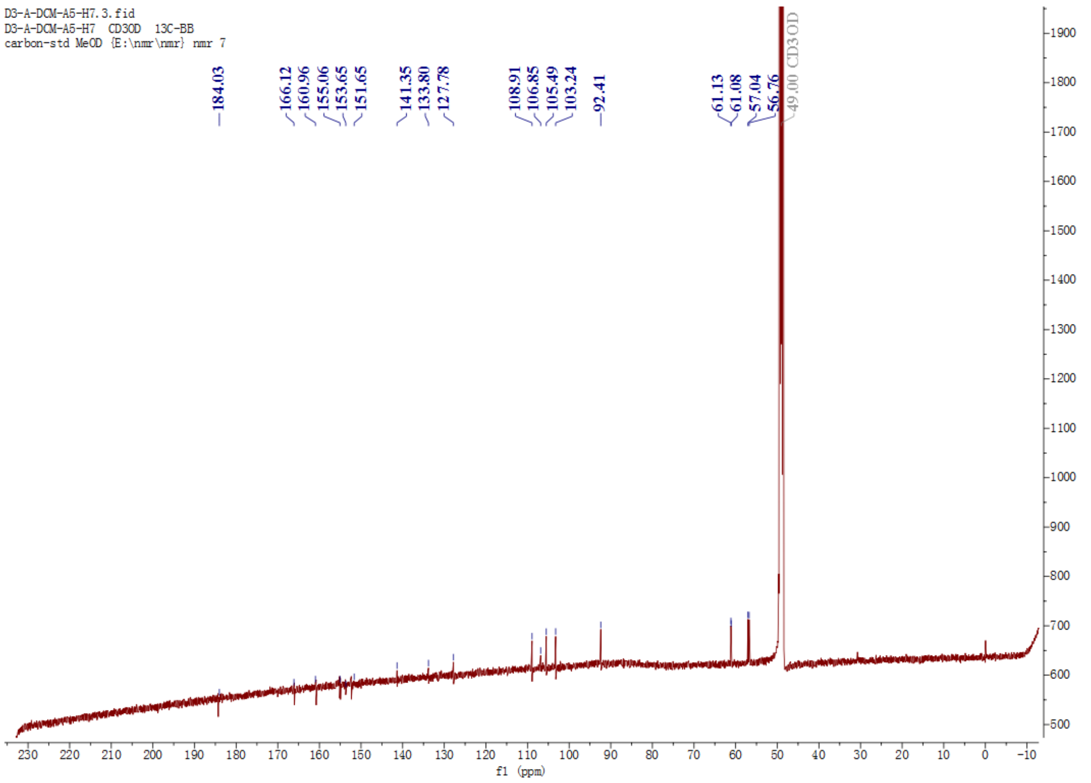

Figure S24. ^{13}C NMR spectrum of compound **6** (150 MHz, CD_3OD)

7. Spectral data of compound **7**

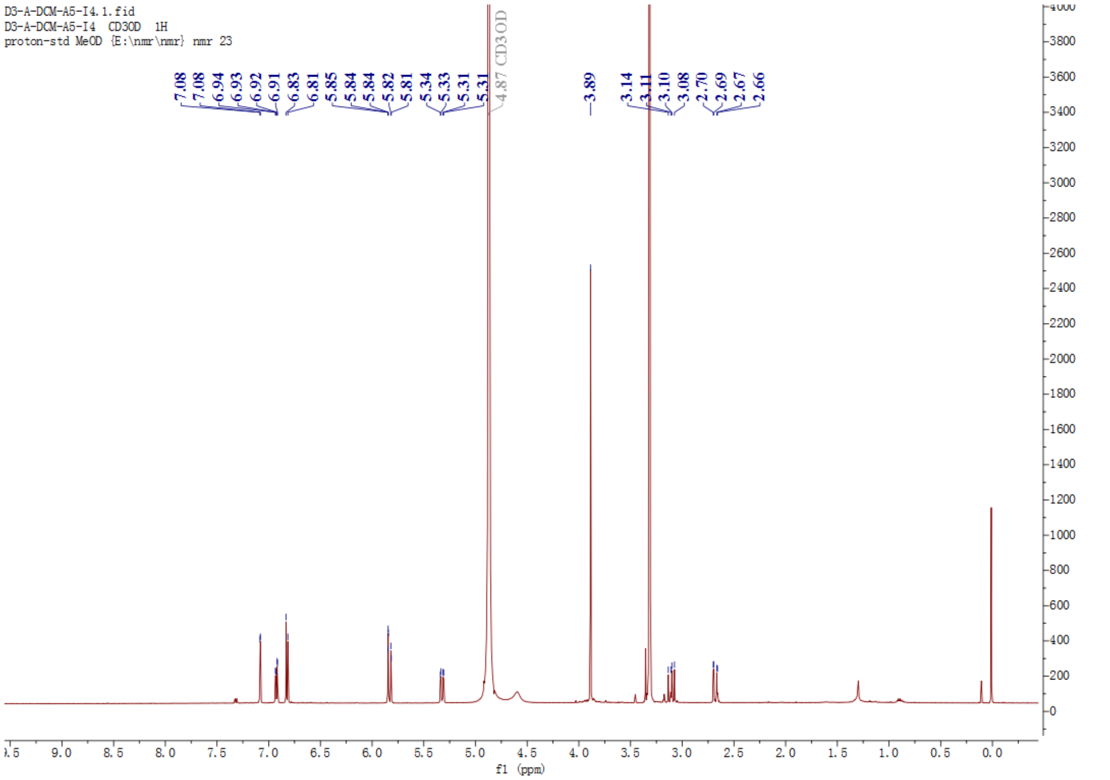

Figure S25. ^{1}H NMR spectrum of compound **7** (600 MHz, CD_3OD)

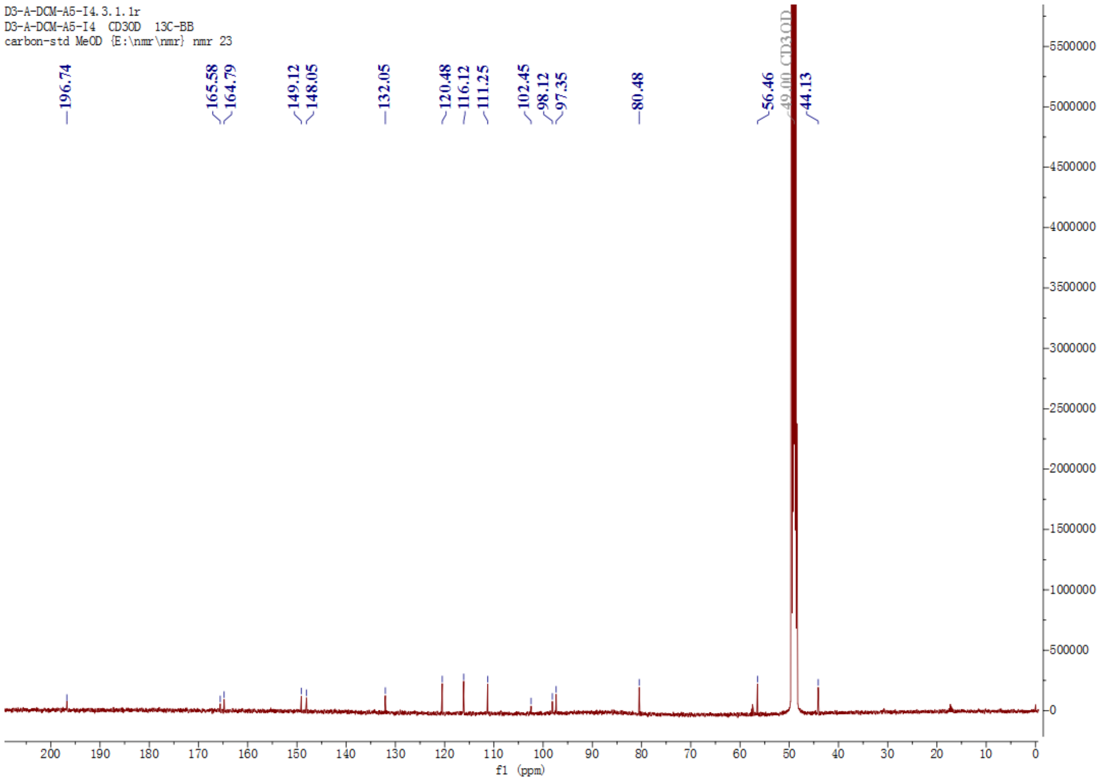

Figure S26. ^{13}C NMR spectrum of compound **7** (150 MHz, CD_3OD)

8. Spectral data of compound **8**

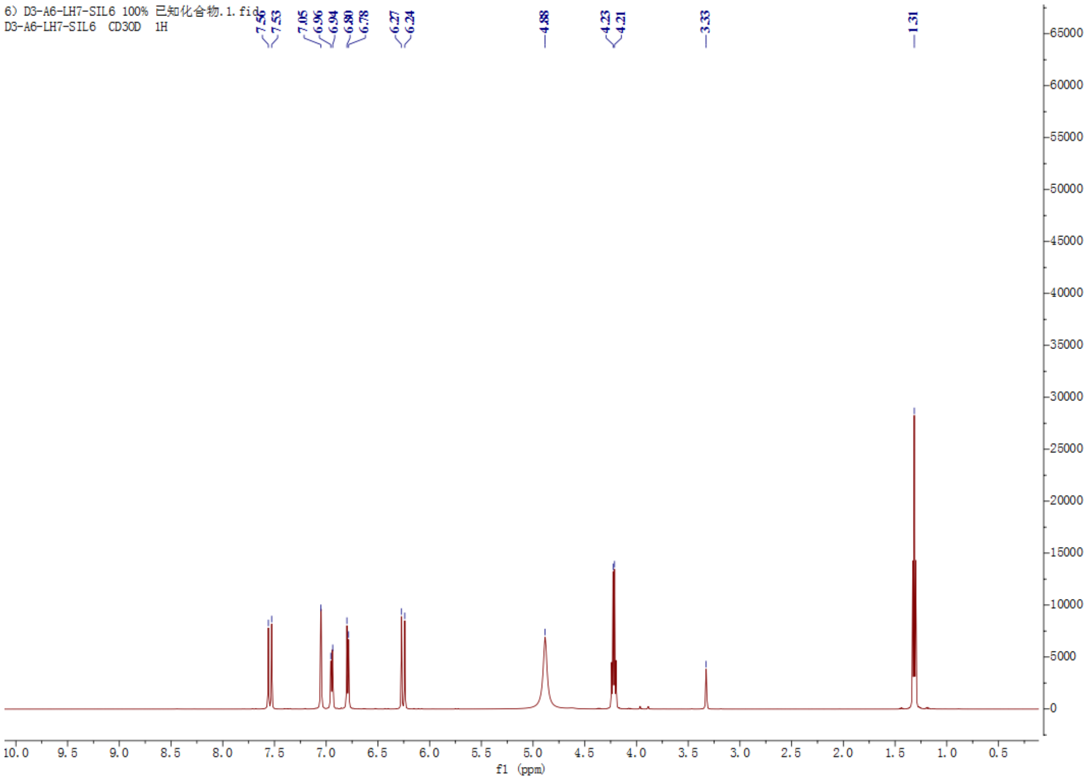

Figure S27. ^{1}H NMR spectrum of compound **8** (600 MHz, CD_3OD)

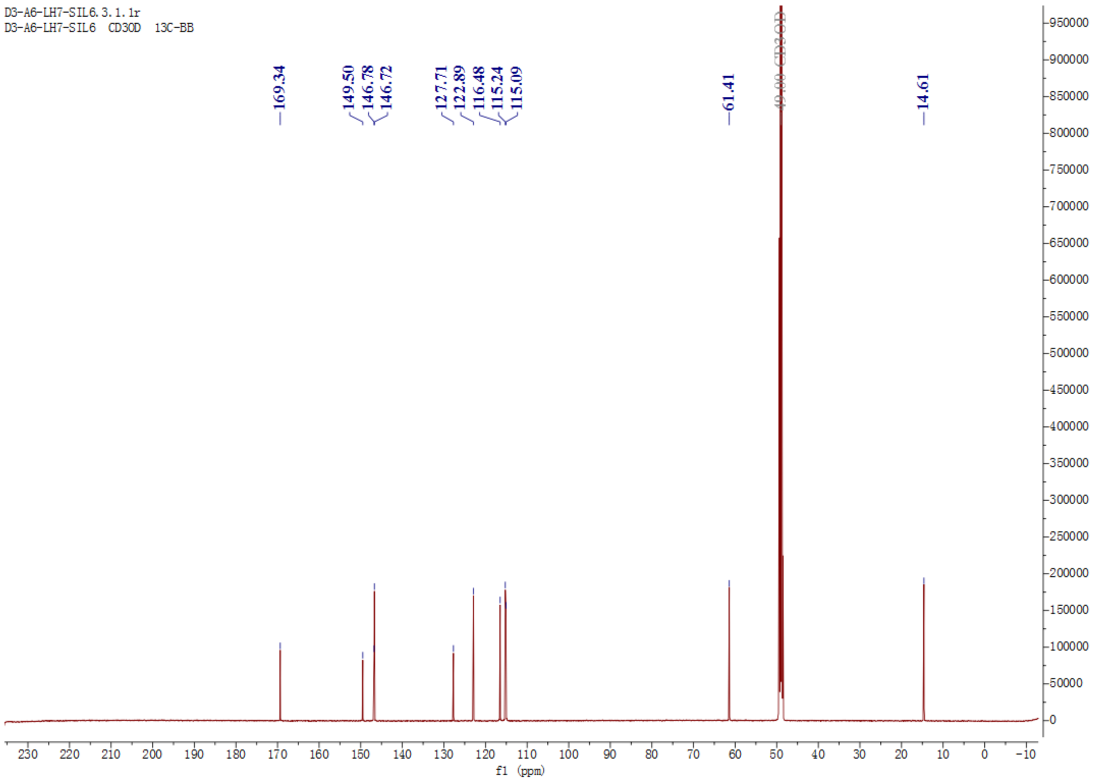

Figure S28. ^{13}C NMR spectrum of compound **8** (150 MHz, CD_3OD)

9. Spectral data of compound **9**

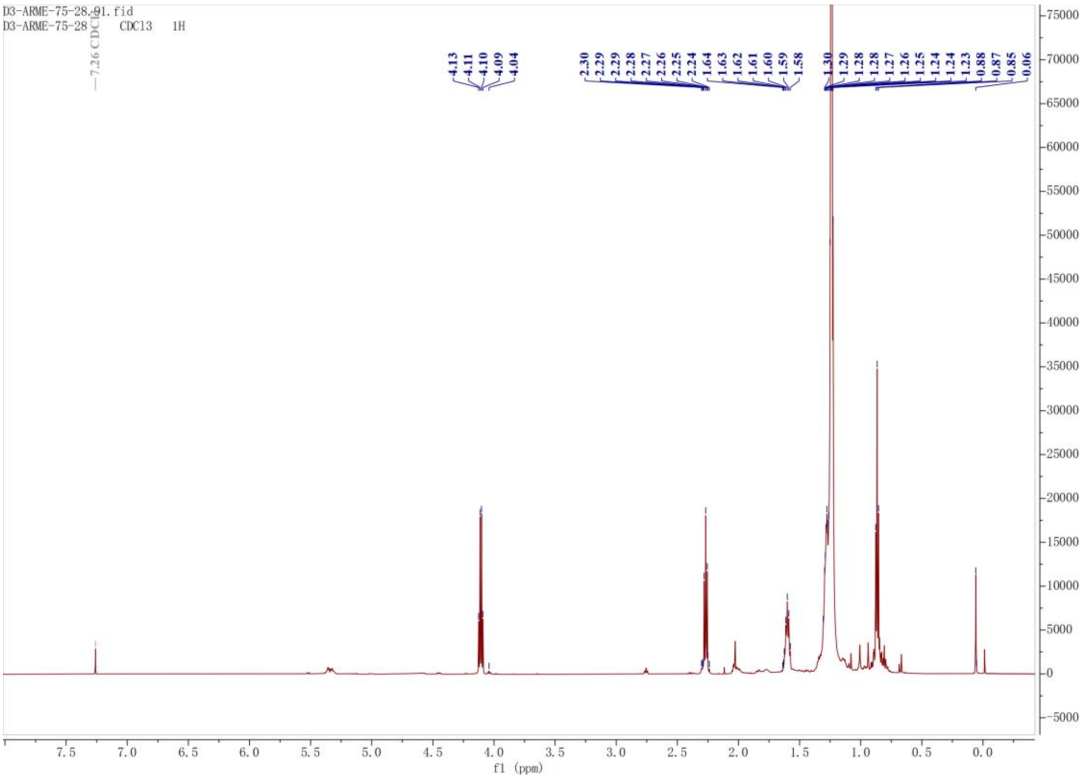

Figure S29. ^{1}H NMR spectrum of compound **9** (600 MHz, CD_3OD)

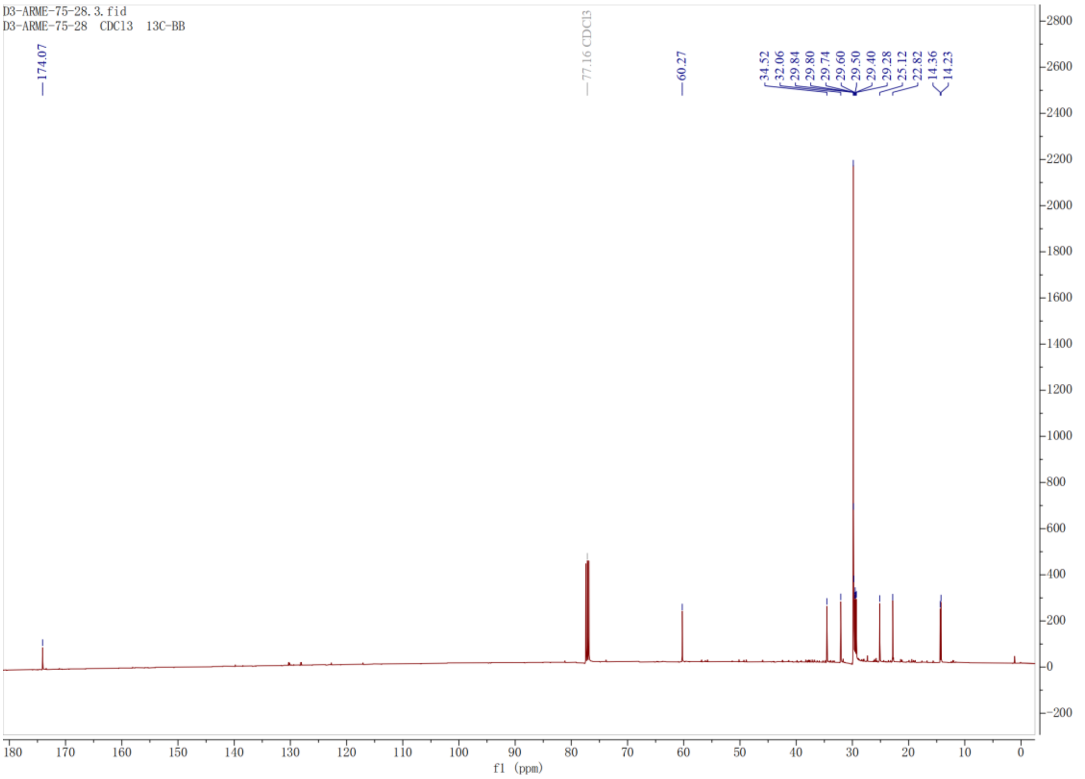

Figure S30. ^{13}C NMR spectrum of compound **9** (150 MHz, CD_3OD)

10. Spectral data of compound **10**

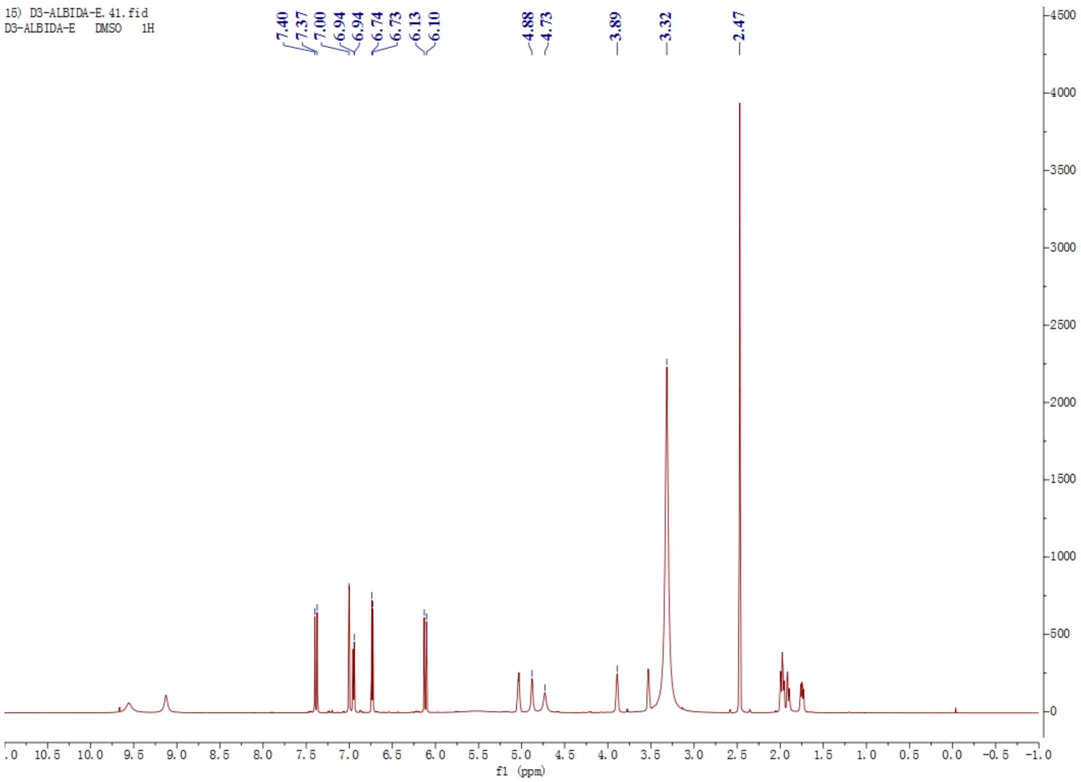

Figure S31. ^{1}H NMR spectrum of compound **10** (600 MHz, CD_3OD)

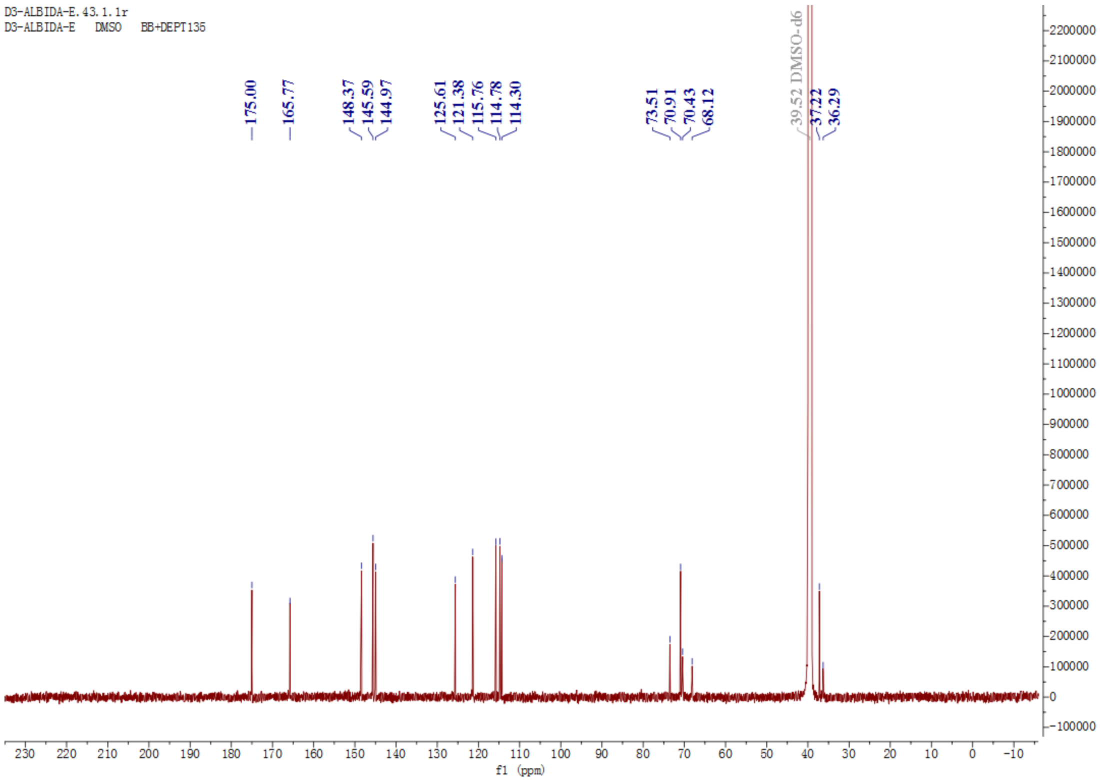

Figure S32. ^{13}C NMR spectrum of compound **10** (150 MHz, CD_3OD)

11. Spectral data of compound **11**

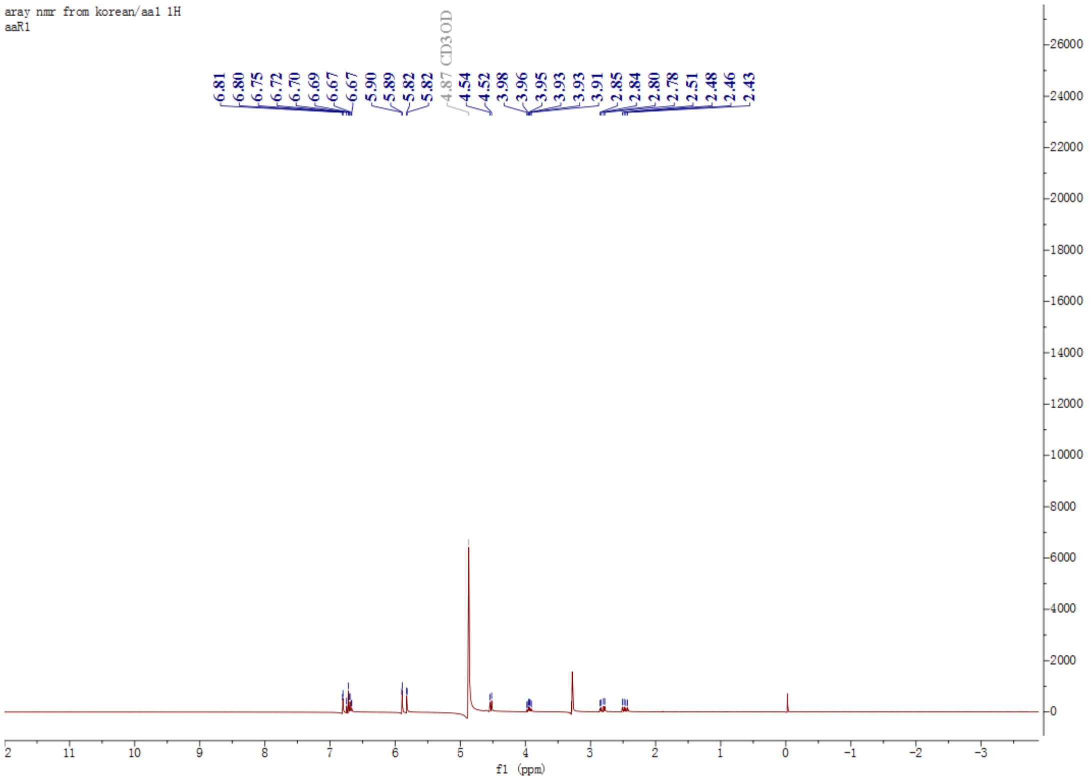

Figure S33. ^{1}H NMR spectrum of compound **11** (600 MHz, CD_3OD)

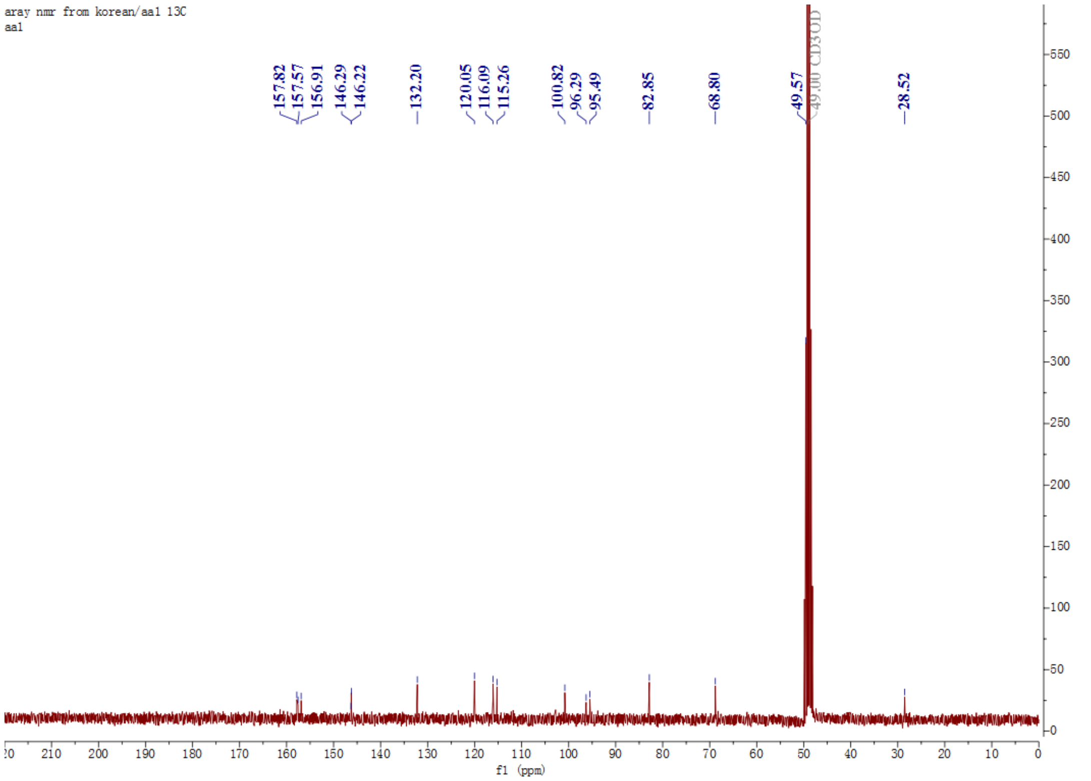

Figure S34. ^{13}C NMR spectrum of compound **11** (150 MHz, CD_3OD)

12. Spectral data of compound **12**

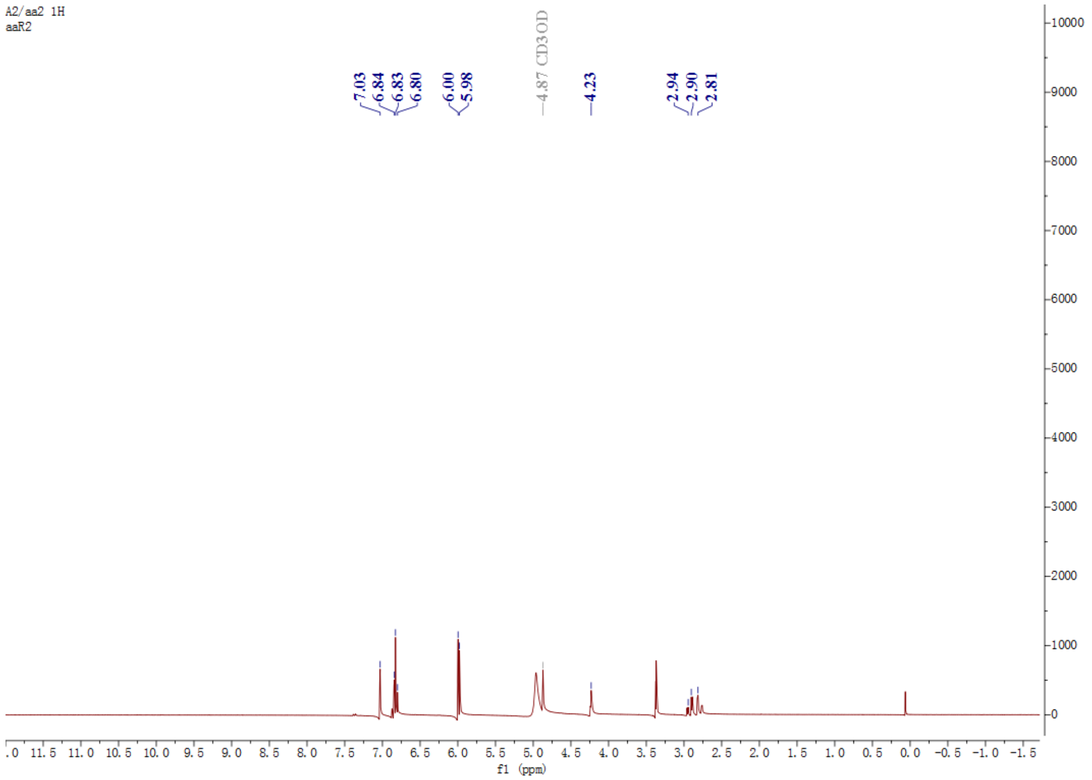

Figure S35. ^{1}H NMR spectrum of compound **12** (600 MHz, CD_3OD)

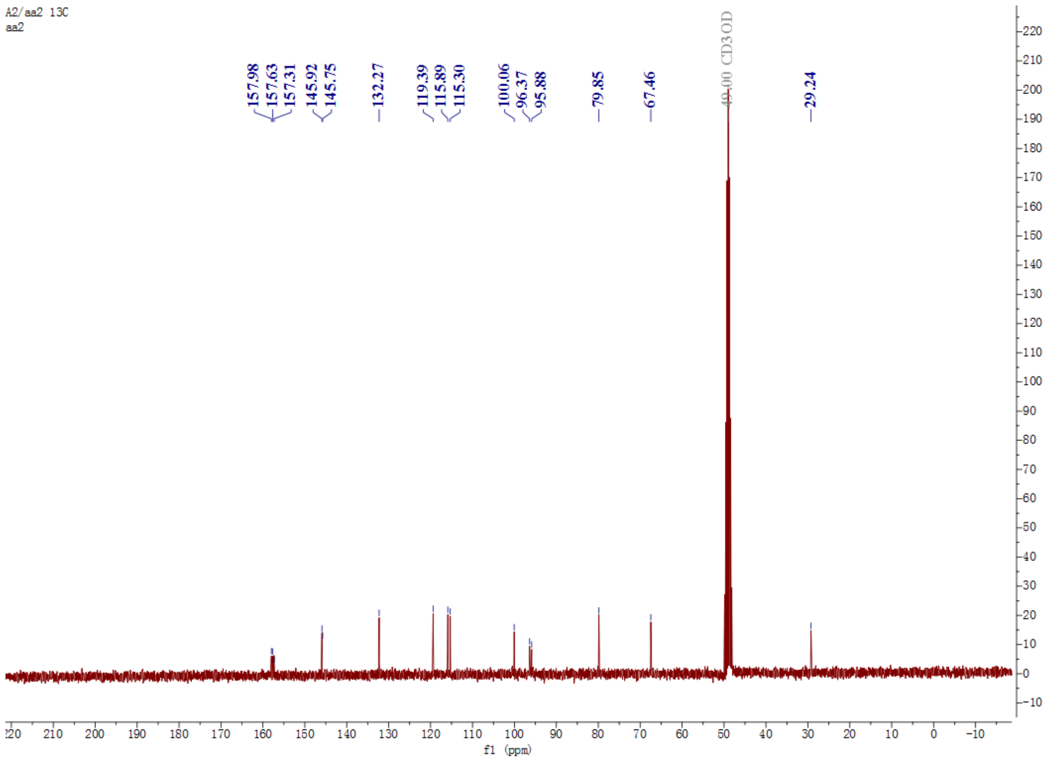

Figure S36. ^{13}C NMR spectrum of compound **12** (150 MHz, CD_3OD)

13. Spectral data of compound **13**

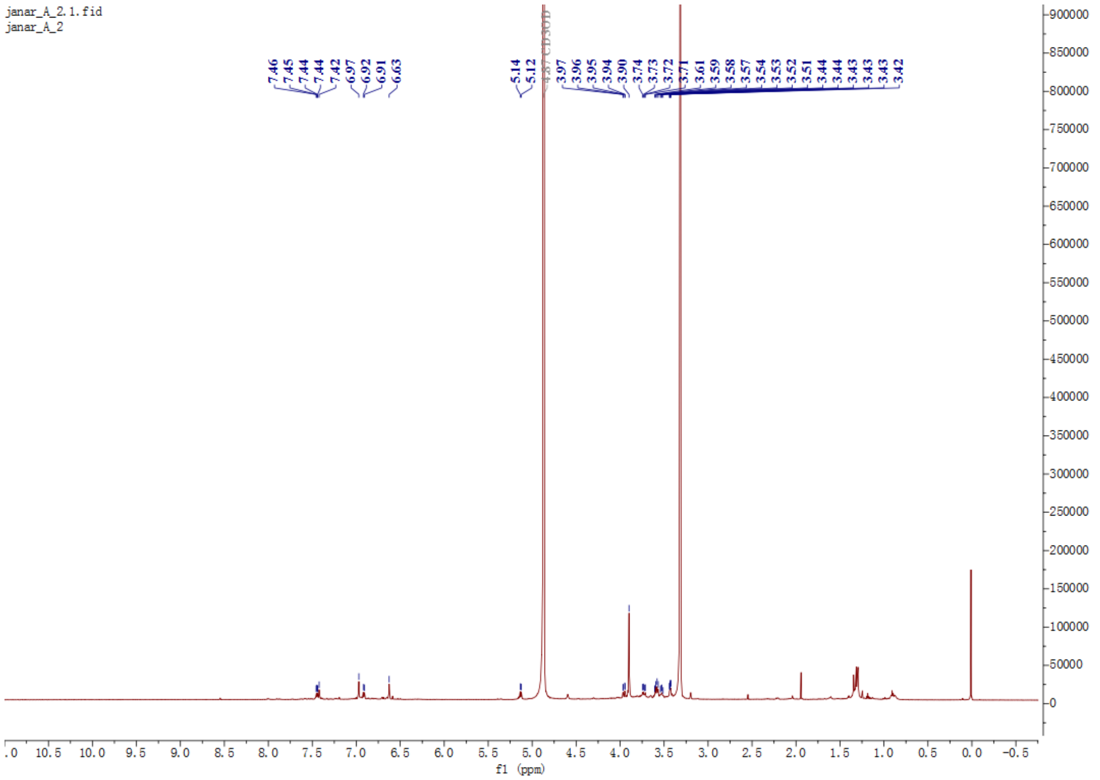

Figure S37. ^{1}H NMR spectrum of compound **13** (600 MHz, CD_3OD)

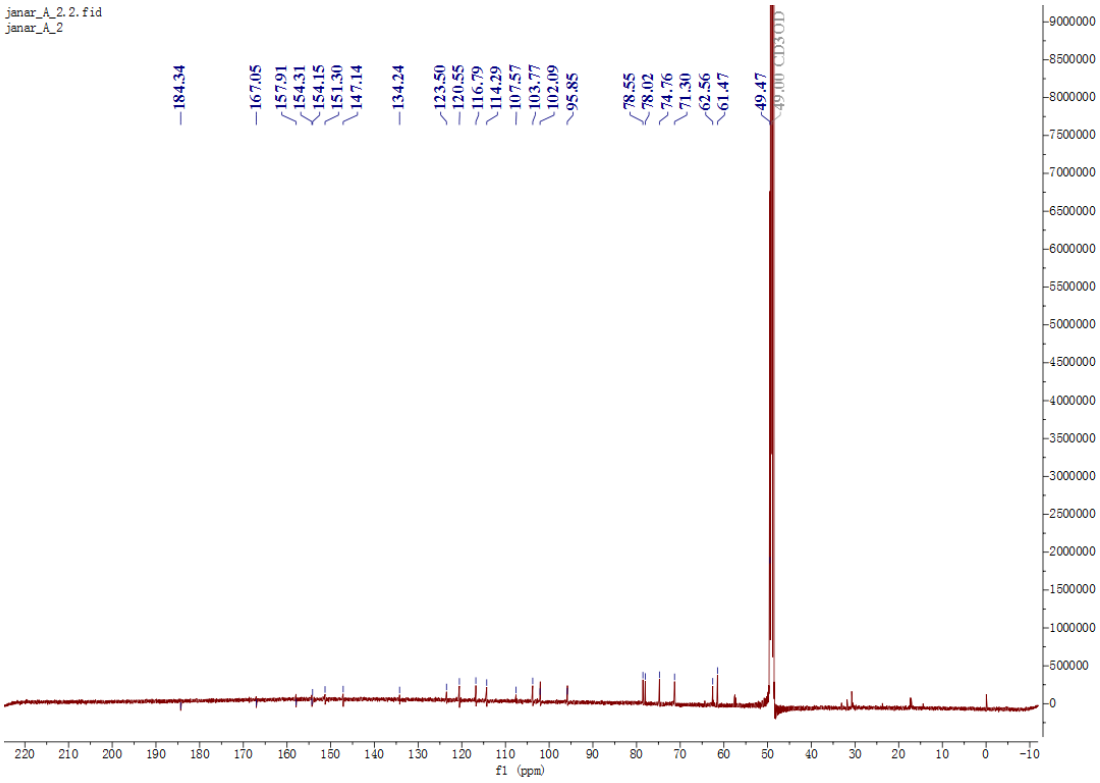

Figure S38. ^{13}C NMR spectrum of compound **13** (150 MHz, CD_3OD)

14. Spectral data of compound **14**

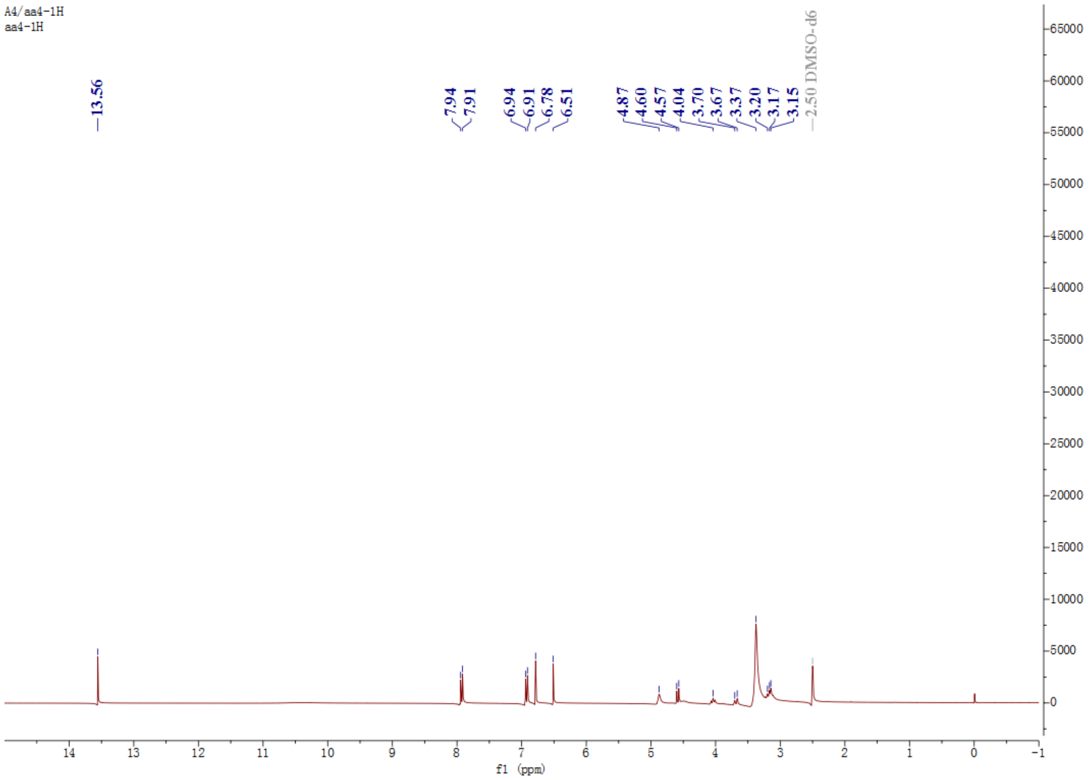

Figure S39. ^{1}H NMR spectrum of compound **14** (600 MHz, CD_3OD)

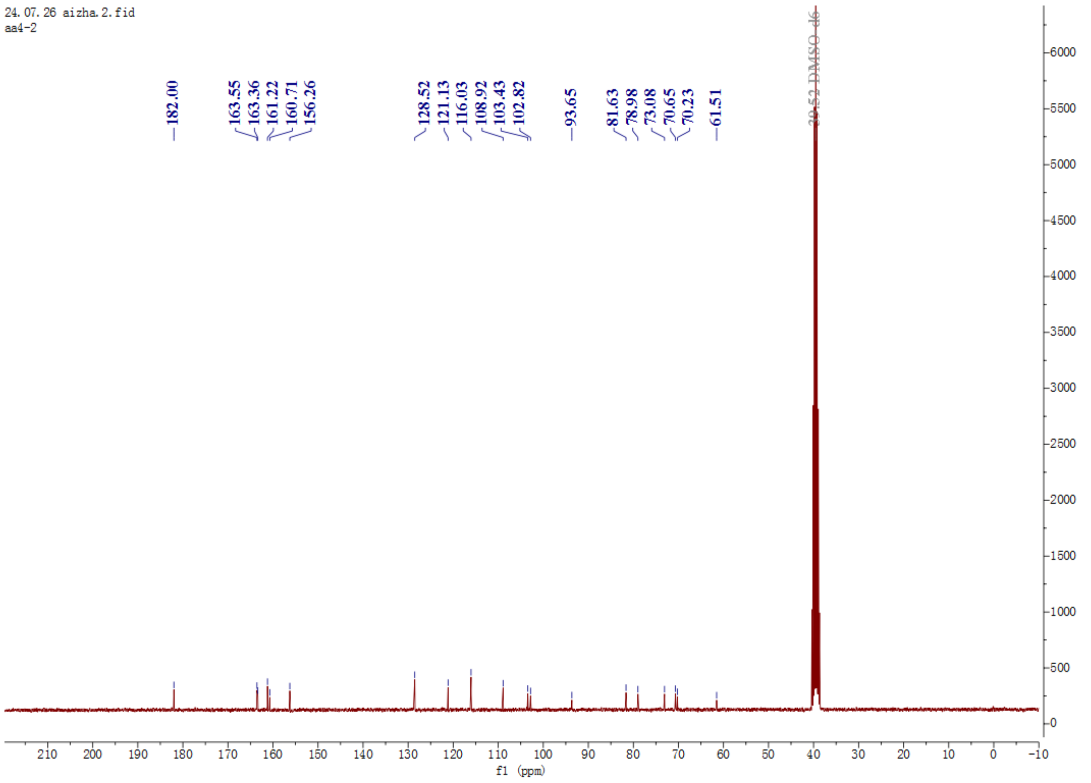

Figure S40. ^{13}C NMR spectrum of compound **14** (150 MHz, CD_3OD)

15. Spectral data of compound **15**

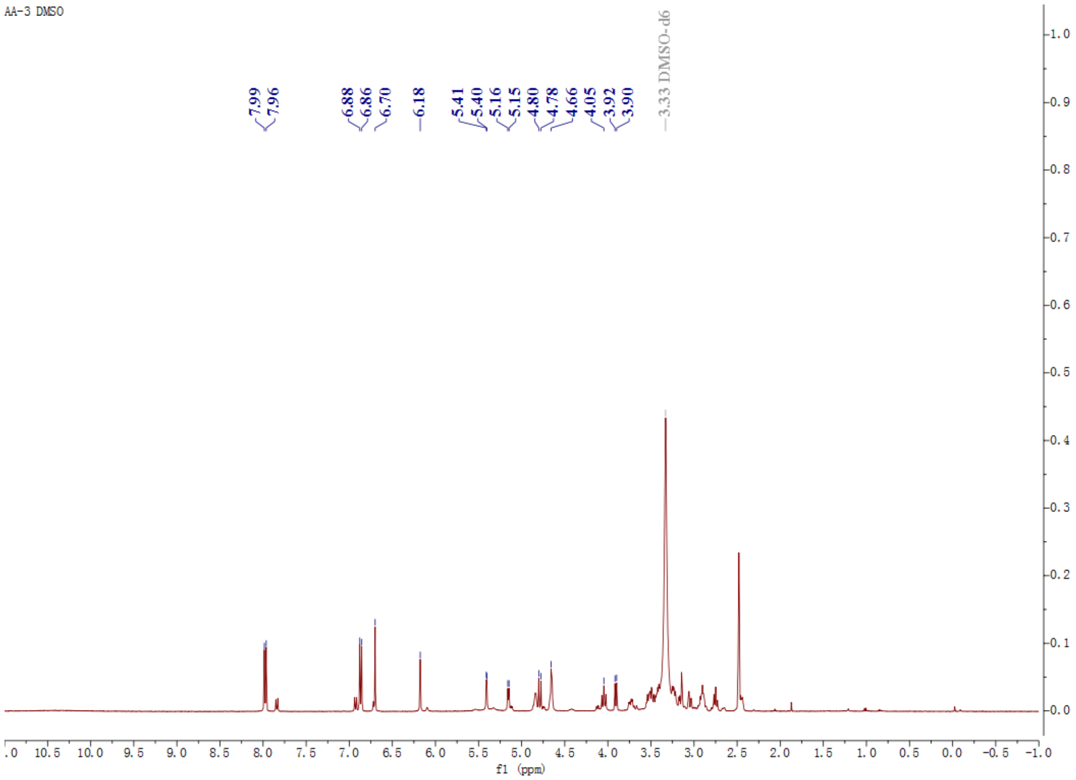

Figure S41. ^{1}H NMR spectrum of compound **15** (600 MHz, DMSO-d_6)

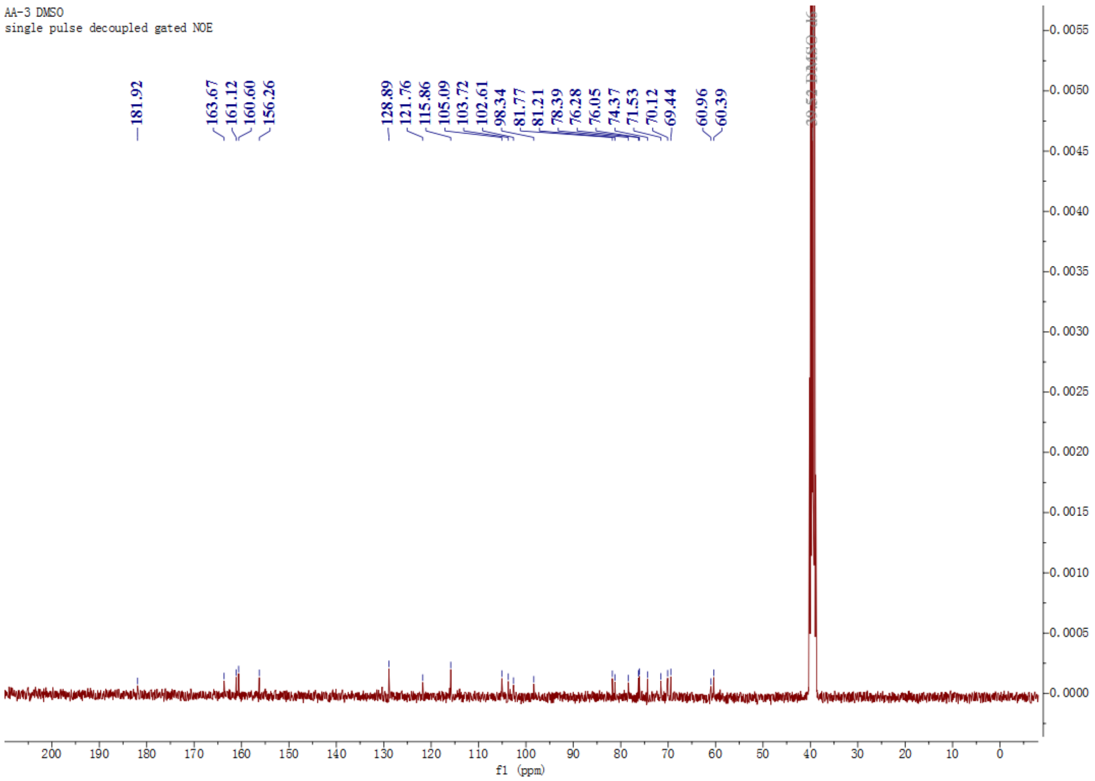

Figure S42. ^{13}C NMR spectrum of compound **15** (150 MHz, DMSO-d_6)

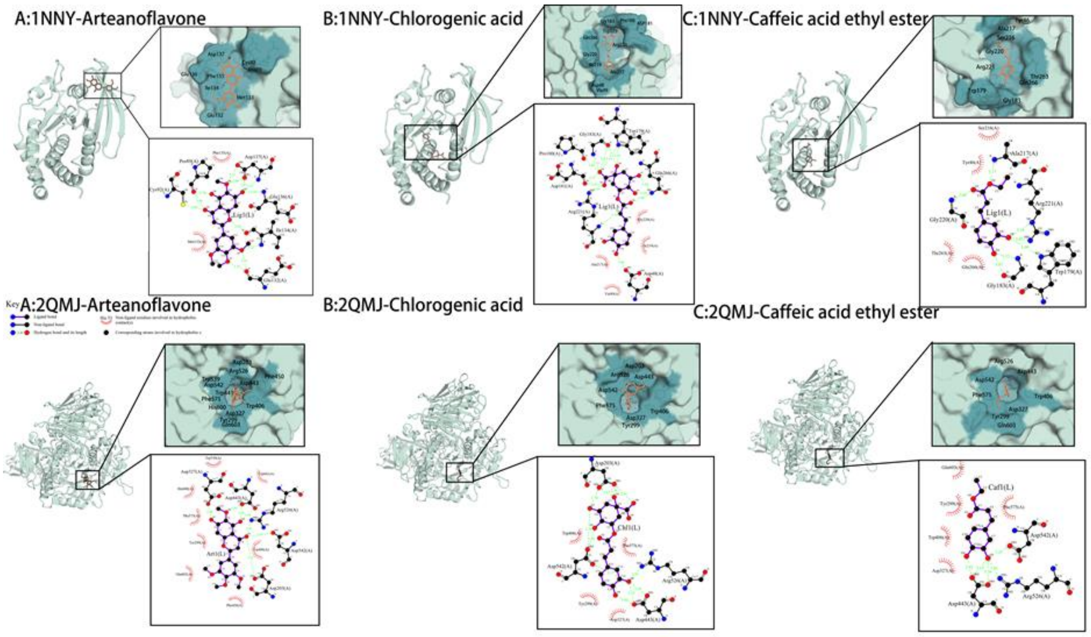

Figure S43. Molecular docking poses of test compounds with (A-C) PTP1B (1NNY) and (D-F) human α glucosidase (2QMJ). (A, D) Arteanoflavone; (B, E) Chlorogenic acid; (C, F) Caffeic acid ethyl ester. Hydrogen bonds are depicted as dashed yellow lines; π-π stacking and cation-π interactions are indicated by green arrows. Key interacting residues are labeled.

Table S1. Summary of conformers selected for re-optimization and their relative free energies and Boltzmann populations.

Conformers	ΔG (kcal/mol)	Boltzmann population	Re-optimized conformations
1	0.00	20.52%	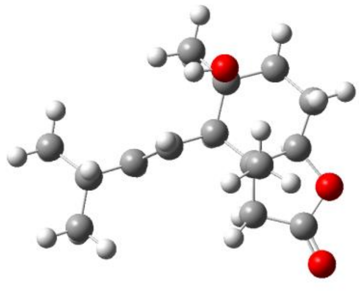
2	0.09	17.51%	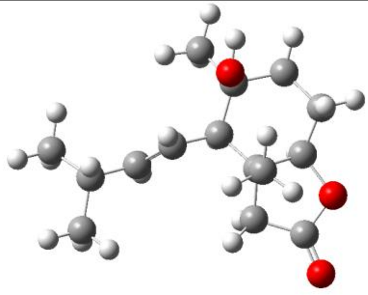

3	0.09	17.50%	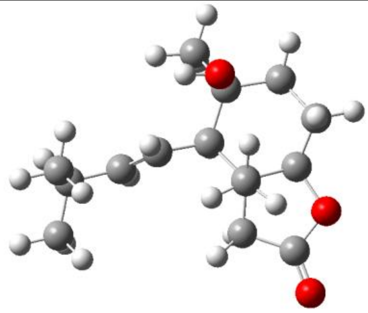
4	0.13	16.34%	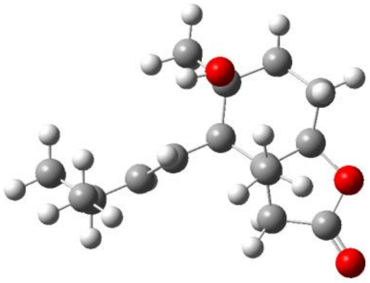
5	0.20	14.59%	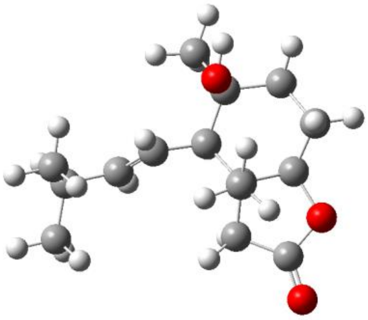
6	0.25	13.55%	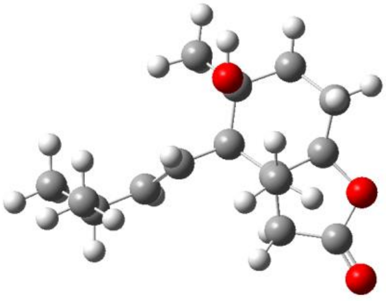
